# Comparative fiber property and transcriptome analyses reveal key genes potentially related to high fiber strength in cotton (*Gossypium hirsutum* L.) line MD52ne

**DOI:** 10.1186/s12870-016-0727-2

**Published:** 2016-02-01

**Authors:** Md S. Islam, David D. Fang, Gregory N. Thyssen, Chris D. Delhom, Yongliang Liu, Hee Jin Kim

**Affiliations:** USDA-ARS, Southern Regional Research Center, Cotton Fiber Bioscience Research Unit, New Orleans, LA 70124 USA; USDA-ARS, Southern Regional Research Center, Cotton Chemistry and Utilization Research Unit, New Orleans, LA 70124 USA; USDA-ARS, Southern Regional Research Center, Cotton Structure and Quality Research Unit, New Orleans, LA 70124 USA

**Keywords:** Bundle fiber strength, Cellulose assembly, Ethylene, *Gossypium hirsutum*, Individual fiber strength, Near-isogenic line, Receptor-like kinase

## Abstract

**Background:**

Individual fiber strength is an important quality attribute that greatly influences the strength of the yarn spun from cotton fibers. Fiber strength is usually measured from bundles of fibers due to the difficulty of reliably measuring strength from individual cotton fibers. However, bundle fiber strength (BFS) is not always correlated with yarn strength since it is affected by multiple fiber properties involved in fiber-to-fiber interactions within a bundle in addition to the individual fiber strength. Molecular mechanisms responsible for regulating individual fiber strength remain unknown. *Gossypium hirsutum* near isogenic lines (NILs), MD52ne and MD90ne showing variations in BFS provide an opportunity for dissecting the regulatory mechanisms involved in individual fiber strength.

**Results:**

Comprehensive fiber property analyses of the NILs revealed that the superior bundle strength of MD52ne fibers resulted from high individual fiber strength with minor contributions from greater fiber length. Comparative transcriptome analyses of the NILs showed that the superior bundle strength of MD52ne fibers was potentially related to two signaling pathways: one is ethylene and the interconnected phytohormonal pathways that are involved in cotton fiber elongation, and the other is receptor-like kinases (RLKs) signaling pathways that are involved in maintaining cell wall integrity. Multiple *RLK*s were differentially expressed in MD52ne fibers and localized in genomic regions encompassing the strength quantitative trait loci (QTLs). Several candidate genes involved in crystalline cellulose assembly were also up-regulated in MD52ne fibers while the secondary cell wall was produced.

**Conclusion:**

Comparative phenotypic and transcriptomic analyses revealed differential expressions of the genes involved in crystalline cellulose assembly, ethylene and RLK signaling pathways between the MD52ne and MD90ne developing fibers. Ethylene and its phytohormonal network might promote the elongation of MD52ne fibers and indirectly contribute to the bundle strength by potentially improving fiber-to-fiber interactions. RLKs that were suggested to mediate a coordination of cell elongation and SCW biosynthesis in other plants might be candidate genes for regulating cotton fiber cell wall assembly and strength.

**Electronic supplementary material:**

The online version of this article (doi:10.1186/s12870-016-0727-2) contains supplementary material, which is available to authorized users.

## Background

Cotton (*Gossypium* spp.) fiber is the most important natural fiber in the textile industry [1]. Physical properties such as strength, length, maturity (degree of thickness), and fineness determine the value and quality of cotton fibers and the yarn spun from them. With the advent of modern high speed spinning machinery, which produces a quality yarn in a cost effective way, the demand for stronger fiber has increased in the highly competitive global textile market. During the past 2 decades, the fiber strength of US cotton has gradually improved through breeding [2], however, the pace of improvement is restricted by our limited knowledge of fiber strength development.

Both cotton producers and processers usually measure fiber strength from a bundle composed of thousands of individual fibers. The strength simply refers to the force required to break fibers [1, 3]. The bundle fiber strength (BFS), which is also called fiber tenacity, is measured by an automated high-volume instrument (HVI) designed to measure the market value of cotton fibers in efficient and rapid ways. Currently, the Agricultural Marketing Service (AMS) of the United States Department of Agriculture (USDA) separates cotton fibers into different classes ranging from the lowest (23.0 g/tex or below) to the highest class (31.0 g/tex and above) based on the BFS values measured by the HVI. A stelometer is used to measure the BFS value for a relatively small amount of fibers [1, 3–5]. Due to the inherent variability among fibers within a bundle, two fiber bundles of the same weight do not have the same number of fibers. Thus, the actual BFS is determined in grams per tex (g/tex) in which the breaking force (g) is standardized by the linear density or fineness (tex = g/km). Hence, high BFS (g/ tex) is obtained by either increasing the breaking force (g) or deceasing the fineness value (tex).

The cotton industry has been using fiber properties determined by the HVI and Advanced Fiber Information System (AFIS) as parameters for predicting yarn quality and selecting the right raw cotton materials to produce different qualities of yarns. However, several textile papers have reported that the BFS data of cotton fibers were inadequate to predict yarn strength due to insignificant correlation between the cotton BFS and the yarn strength [4, 6, 7]. The BFS values are regulated by intrinsic fiber strength and other fiber properties which affect fiber-to-fiber interactions within a bundle [1, 3, 4, 8–10]. Fiber length values that promote fiber interactions within a bundle impact the BFS values [11]. Fiber thickness-related properties including micronaire (MIC), maturity ratio (MR), and fineness values (tex) also affect bundle strength of cotton fibers since they determine the number of individual fibers in a fiber bundle [9].

The intrinsic fiber breaking force value is correlated with yarn strength and can be measured from individual fibers by Mantis or Favimat instruments [1, 3, 8, 9, 11]. Average breaking force (cN) obtained from several hundred individual fibers was not affected by other bundle fiber properties and was reported as one of the most important factors in determining the strength of the yarn spun from those fibers [4, 12, 13]. Despite the usefulness of the individual fiber properties, the Mantis and Favimat instruments, which require laborious processes, have not been previously utilized in cotton genetics and genomics research.

*Gossypium hirsutum* germplasm NILs, MD52ne and MD90ne were developed through backcross breeding [14]. MD90ne is the recurrent parent and MD52ne is a BC_6_ high-BFS selection. MD52ne contains 10 % higher BFS values, 22 % less short fibers, and 7 % greater fiber length than its NIL, MD90ne [15]. The stronger BFS trait of MD52ne was suggested to be controlled by a small number (≤2) of genes [15]. By using the prototype of cotton oligonucleotide microarray chips that was developed from cotton expressed sequence tags (ESTs) [16], we previously observed a temporal up-regulation of secondary cell wall (SCW) biogenesis genes in MD52ne fiber at the transition from fiber elongation to secondary cell wall (SCW) biosynthesis as compared with MD90ne [17]. Another group also suggested that SCW biogenesis-related genes might contribute to the fiber strength of *G. hirsutum* chromosome introgression lines (CSILs) carrying chromosomal segments from *G. barbadense* whose fibers are longer, stronger, and finer than their recurrent parent *G. hirsutum* TM-1 [18]. The *G. hirsutum* CSILs were used for identifying potential genes responsible for fiber length [19] in addition to fiber strength [18]. Since the fiber length and fineness affecting fiber-to-fiber interactions within a bundle can modulate the BFS values of the cotton NILs [17] and CSILs [18], it is not clear if the SCW biogenesis-related genes are involved in regulating individual fiber breaking force and/or other bundle fiber properties. Indeed, the same SCW biogenesis-related genes were identified as differentially expressed genes (DEGs) that may be involved in molecular mechanisms of controlling fiber length [19] and fiber thickness-related properties [20, 21] in addition to the BFS [17, 18]. Therefore, it remains to be answered which candidate genes are really involved in individual fiber breaking force and therefore yarn strength.

In this research, we measured fiber properties of mature and developing fibers of MD52ne, MD90ne, and their F_2_ progenies using both novel and conventional methods including Favimat, cross-section image analysis microscopy, attenuated total reflectance Fourier transform infrared spectroscopy (ATR-FTIR), HVI, Stelometer, AFIS, and gravimetric fineness methods. The extended fiber property analyses showed that the superior BFS of MD52ne fibers resulted primarily from a higher individual fiber breaking force with a minor contribution from increased fiber length. Comparative transcriptome analyses of the NILs suggested that the superior BFS of MD52ne fibers was potentially related to ethylene pathway-directed fiber elongation and enhanced cell wall integrity due to the activity of receptor-like kinase (RLK) signaling pathways.

## Results

### Comparisons of bundle fiber properties between mature MD52ne and MD90ne fibers

Fiber property analysis by HVI showed that the BFS of mature MD52ne fibers was significantly greater (19 ~ 25 %) than that of MD90ne fibers grown in two separate fields that substantially differ in geographic location and environmental conditions (Table 1). In addition, upper half mean length (UHML) of the MD52ne fiber was longer (5 ~ 6 %). Average fiber elongation values of MD52ne were lower than those of MD90ne but was only statistical significant at one location. No significant differences in the MIC values were observed between the MD52ne and MD90ne grown in both locations.

**Table 1:** Comparisons of HVI bundle fiber properties between MD52ne and MD90ne

Fiber properties	Cotton Fields	MD52ne	MD90ne	MD52ne/MD90ne	*t* test
					*p* value
BFS (g/tex)	Stoneville, MS	39.95 ± 1.84	33.61 ± 1.61	1.19	<0.0001***
	New Orleans, LA	39.69 ± 2.69	31.79 ± 2.87	1.25	0.002**
UHML (mm)	Stoneville, MS	31.24 ± 0.51	29.72 ± 1.02	1.05	< 0.0005***
	New Orleans, LA	31.50 ± 0.76	29.85 ± 0.51	1.06	0.0059**
FE (%)	Stoneville, MS	5.45 ± 0.18	5.96 ± 0.26	0.91	< 0.0001***
	New Orleans, LA	4.74 ± 0.13	4.94 ± 0.60	0.96	0.487
MIC	Stoneville, MS	4.94 ± 0.29	5.01 ± 0.07	0.99	0.468
	New Orleans, LA	5.86 ± 0.79	5.25 ± 0.23	1.11	0.136

Each value is the mean ± SD. Statistical significance was shown at the probability levels under 0.01**, and 0.001***. HVI, High Volume Instrument; BFS, bundle fiber strength; UHML, upper half mean length; UI, uniformity index; FE, fiber elongation; MIC, micronaire

### Correlation of bundle fiber strength with other fiber properties

**Fig. 1:**
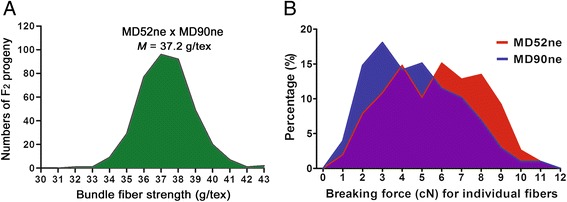
Fiber property analyses of NILs and their F_2_ progeny. **a** Frequency distribution of bundle fiber strength in an F_2_ population from a cross between MD90ne and MD52ne. Average bundle strength value of each F_2_ progeny was obtained from five replicates by HVI. Minimum, median (*M*), and maximum bundle fiber strengths were 31.99, 37.22, and 42.66 g/tex among 384 F_2_ progenies. **b** Comparisons of breaking force distributions of individual fibers from MD52ne and MD90ne fibers. Breaking forces (cN) of 303 individual fibers were determined from mature MD52ne and MD90ne fibers having similar fiber maturity with an identical MIC value (4.931). A 13 mm length gauge was used for breaking fiber with Favimat instrument

To determine how the BFS values of the NILs were affected by other physical properties involved in fiber-to-fiber interactions, we developed an F_2_ population of 384 progeny plants derived from a cross between MD52ne and MD90ne. Among the F_2_ progenies, the BFS values showed a Gaussian distribution with a broad range (31.99 and 42.66 g/tex) (Fig. 1a). Other fiber properties including UHML length (27.82 ~ 32.48 mm), elongation (4.78 ~ 6.45 %), MIC (4.39 ~ 5.78), maturity ratio (0.979 ~ 1.078), and fineness (172.0 ~ 215.0 mtex) measured by HVI and AFIS showed Gaussian distributions among the F_2_ progenies so we were able to determine correlations of the BFS with other fiber physical properties.

Table 2 showed that the BFS values of the NILs were affected by fiber length (UHML, mm) and micronaire (MIC) measured by HVI, and the maturity ratio (MR) and fineness (mtex) measured by AFIS. The correlation coefficient values (*r*) by Pearson’s method [22] showed that the BFS value was positively correlated with UHML, MIC, MR, or fineness, whereas the BFS showed no significant correlation with FE. The *R*^*2*^ values suggested that BFS variances were slightly affected by UHML (12.9 %), whereas they had little effect from fiber thickness-related properties such as MIC (1.2 %), MR (2.9 %), and fineness (1.2 %).

**Table 2:** Correlations between bundle fiber strength and other fiber properties from the F_2_ plant derived from a cross between MD52ne and MD90ne

Fiber properties	Measuring method	Sample numbers	Correlation coefficient (*r*)	*R* ^*2*^	*p*-value	Correlation with BFS
UHML	HVI	384	0.360	0.129	<0.0001***	Yes
MIC	HVI	384	0.108	0.012	0.0343*	Yes
MR	AFIS	380	0.169	0.029	0.001***	Yes
Fineness	AFIS	380	0.110	0.012	0.0325*	Yes
FE	HVI	384	−0.049	0.002	0.340	No

Average bundle fiber strength (BFS), fiber length (UHML), micronaire (MIC), maturity ratio (MR), fineness, and fiber elongation (FE) were calculated from five replicates of each F_2_ progeny plant. Linear regression and correlation data between bundle fiber strength and other fiber properties were determined by Graphad Prism 5.0. Statistical significance was shown at the probability levels under 0.05*, and 0.001***

### Comparisons of individual fiber properties between mature MD52n and MD90ne fibers

To minimize the influences of other physical fiber properties on fiber strength, we first screened for the NIL plants that had similar fiber properties except the BFS values. Among the selected NIL plants having a significant variation (24 %) of BFS values, we identified each cotton MD52ne and MD90ne line having an almost identical MIC value (4.931) representing a combination of fiber maturity ratio and fineness. The fiber properties measured by AFIS in addition to the HVI confirmed that there was no significant variation in the fiber thickness-related properties (MIC, MR, and fineness) but a significant difference (5 %, UHML) in fiber length between the selected NILs (Table 3). The average breaking force of individual MD52ne fibers was significantly greater (22.4 %) than that of individual MD90ne fibers (Table 3). The distribution curves showed great variation of breaking forces among individual fibers of each NIL and a similar distribution range of breaking force between the individual MD52ne fibers (0.93 ~ 11.25 cN) and MD90ne fibers (0.62 ~ 11.27 cN) (Fig. 1b). Stronger fibers with higher cN values were more prevalent in MD52ne than MD90ne.

**Table 3:** Comparisons of bundle and individual fiber properties between mature MD52ne and MD90ne fibers having identical MIC value

Measuring methods	Fiber Properties	MD52ne	MD90ne	MD52ne/MD90ne	*t* test
					*p* value
HVI	MIC	4.931 ± 0.02	4.931 ± 0.06	1.00	1.0000
	BFS (g/tex)	39.27 ± 1.41	31.66 ± 1.19	1.24	< 0.0001***
	UHML (mm)	31.01 ± 0.79	29.59 ± 0.48	1.05	0.0089**
AFIS	Maturity ratio (MR)	0.99 ± 0.02	0.97 ± 0.01	1.02	0.1963
	Fineness (mtex)	171.67 ± 6.11	173.67 ± 3.06	0.99	0.6389
Favimat^a^	Breaking force (cN)	5.67 ± 2.34	4.63 ± 2.18	1.22	< 0.0001***

^a^Average breaking force (cN) was calculated from 303 individual fibers by Favimat. Each value is the mean ± SD. Statistical significance was shown at the probability levels under 0.05*, 0.01**, and 0.001***. HVI, High Volume Instrument; AFIS, Advanced Fiber Information System; MIC, micronaire; BFS, bundle fiber strength

### Comparisons of fiber properties between developing MD52ne and MD90ne fibers

**Fig. 2:**
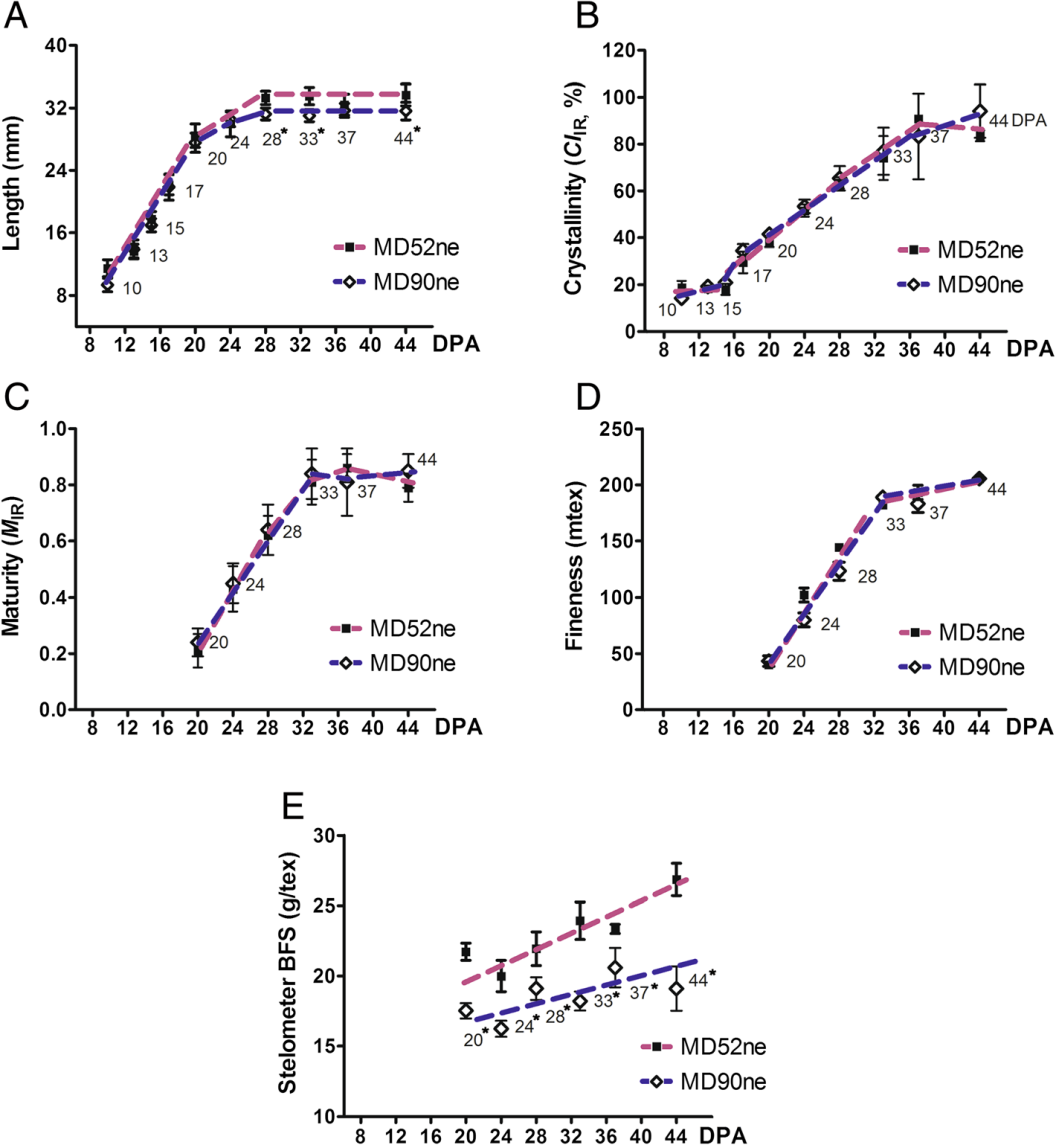
Comparisons of fiber properties from developing MD52ne and MD90ne fibers. Developing fibers at nine different developmental time points (10, 13, 15, 17, 20, 24, 28, 33, and 37 DPA) and full developed fibers at 44 DPA were collected from NILs grown side by side in replicated fields in New Orleans, LA in 2013. Asterisks next to the time points denote statistical significance. **a** Fiber length. Average lengths of developing fibers at different DPAs were calculated from 30 replicates. **b** Crystallinity. Average crystallinity (*CI*_IR_) was determined from six replicates of ATR-FTIR spectra. **c** Maturity. Average fiber maturity (*M*_IR_) was determined from six replicates using ATR-FTIR. **d** Fineness. Three hundred fibers of 15 mm length were used for each replicate of gravimetric fineness analyses. Average fineness values were calculated from three replicates. **e** Bundle fiber strength. Average bundle fiber strength of developing fibers was measured from three replicates by Stelometer

**Fig. 3:**
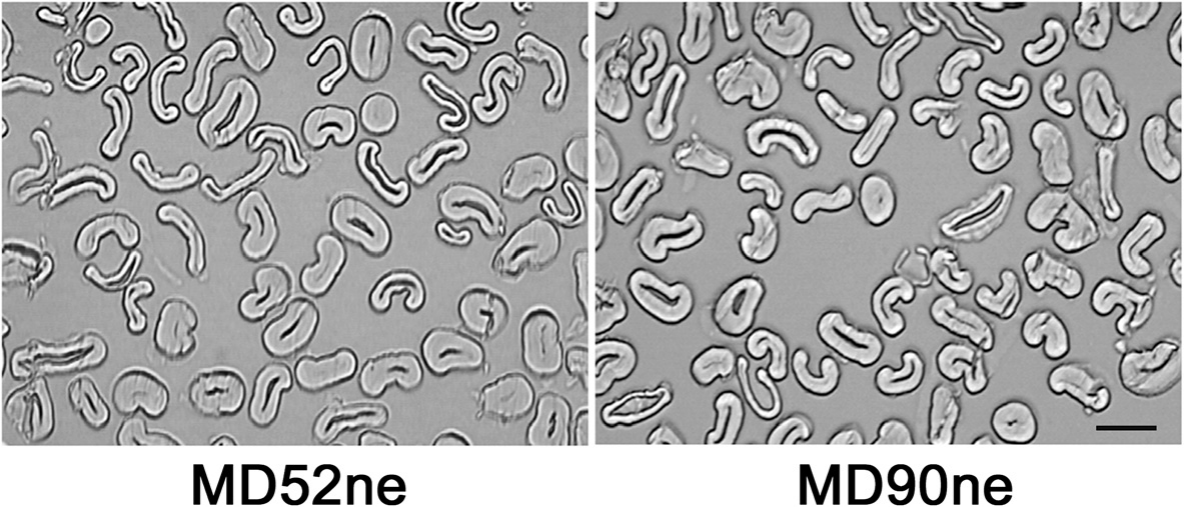
Comparisons of fiber maturity ratios of mature fibers between MD52ne and MD90ne measured by image analysis microscopy. Circularity (θ = 4πA/P^2^) representing the degree of fiber cell wall thickness was calculated from average wall area (A) excluding lumen and perimeter (P) of cross-sections of 300 fibers. The maturity ratios between MD52ne (0.948 ± 0.010) and MD90ne (0.971 ± 0.033) calculated from the circularities showed no significant variation (*p*-value, 0.519). A scale bar represents 10 μm. MD52ne, MD90ne

To determine when variations of fiber properties occurred during cotton fiber development between the two NILs, we measured fiber physical properties from developing fibers at ten different developmental time points (10, 13, 15, 17, 20, 24, 28, 33, 37, and 44 DPA) covering entire developmental stages (Fig. 2). The growth rates of actively elongating MD52ne and MD90ne fibers declined at 20 DPA (Fig. 2a). Statistical analyses using *t* test showed no significant variances on the average lengths of the developing fibers younger than 24 DPA between the NILs. Average fiber lengths of developing MD52ne fibers at 28 DPA and older were significantly longer (*p* value, 0.0012) than those of developing MD90ne fibers at the corresponding time points. At around 15 DPA, fibers entered a transition phase with both SCW cellulose biosynthesis and fiber elongation (Fig. 2b). Crystallinities of developing MD52ne and MD90ne fibers were measured by an ATR-FTIR spectroscopy and its corresponding algorithm [23, 24]. The comparative plot showed low crystallinity (*CI*_IR_) of the developing fibers between 10 and 15 DPA. The *CI*_IR_ values increased rapidly to 17 DPA, and reached its peak at 37 DPA (Fig. 2b). The observation implied that the developing fibers at 10, 13, and 15 DPA were composed of mainly primary cell wall (PCW) containing a low crystallinity and the transition from elongation to SCW biosynthesis occurred from 15 to 17 DPA. SCW containing a high crystallinity was deposited on the developing fibers until 37 DPA of both NILs. There was no statistically significant difference in the *CI*_IR_ values of developing fibers between the two NILs during the entirety of the developmental stages (Fig. 2b). The bolls became fully developed and began to open at approximately 42 DPA. The mature fibers dehisced at 44 DPA. Fiber maturity ratio or degree of wall thickness (*M*_IR_) was also assessed from developing MD52ne and MD90ne fibers by ATR-FTIR spectra (Fig. 2c) [23, 24]. The developing fibers at 17 DPA and younger showed little of the SCW cellulose that is mainly responsible for fiber maturity [25]. The *M*_IR_ values revealed that the developing fibers at 20 DPA and older consisted of SCW cellulose. During the SCW biosynthesis stage (20–33 DPA) of both NIL fibers, the *M*_IR_ values increased likewise (Fig. 2c). Cross-sectioned images of fully mature MD 52ne and 90ne fibers and their calculated MR showed similarities between the NILs (Fig. 3). The increasing pattern of fineness values (40.0 to 205.6 mtex) of developing MD52ne fibers from 20 to 44 DPA was almost identical to that (43.3 to 205.6 mtex) of developing MD90ne fibers at the corresponding time points (Fig. 2d). Developing MD52ne fibers at 20 DPA and older were significantly (*p*-value < 0.0008) stronger than developing MD90ne fibers at the corresponding DPAs (Fig. 2e). When the developing fibers reached 20 DPA, the BFS differences were clearly detected between MD52ne (21.7 g/tex) and MD90ne (17.5 g/tex). A stelometer, which requires relatively less fiber than the HVI, was used for measuring bundle strength of developing fibers. Neither the stelometer nor the HVI could measure BFS values from developing fibers that were younger than 20 DPA, because the sugary and sticky developing fibers cannot be individualized.

### Transcriptome analysis of developing fibers between MD52ne and MD90ne by RNA-seq

To investigate the molecular basis for the superior fiber strength of MD52ne to MD90ne, whole genome comparative transcriptome analyses were performed with total RNAs extracted from developing fibers between MD52ne and MD90ne. Based on the fiber property data obtained from developing fibers (Fig. 2), two different developmental time points, 15 DPA containing mainly PCW and 20 DPA containing both PCW and SCW, were compared between the NILs with two biological replicates.

The average number of raw reads per library obtained by paired-end Illumina sequencing ranged from 32.80 to 33.49,000,000 reads (Table 4). The numbers of average raw reads were slightly higher in MD52ne in both time points than MD90ne. Of the raw reads, 80.83 to 84.35 % of reads per library were mapped to annotated protein coding genes in the draft genome *G. hirsutum,* TM-1 [26]. A total of 61,263 genes were mapped in this Upland cotton genome for all four libraries (Additional file 1). Expressed genes were annotated with Arabidopsis Tair 10 homeolog genes. The percentages of genes having reads per kilo-base per million reads (RPKM) >1 were ranged from 71.61 to 79.22 % per library. We selected an RPKM threshold of 1 to be consistent with prior work using this draft genome [26].

**Table 4:** Summary of total raw and clean sequence tags

Item	MD52ne				MD90ne			
Time point	15 DPA		20 DPA		15 DPA		20DPA	
Replication	Rep. 1	Rep. 2	Rep. 1	Rep. 2	Rep. 1	Rep. 2	Rep. 1	Rep. 2
Total bases (million)	3382.41	3247.35	3187.87	3382.75	3347.42	3313.08	3384.49	3096.28
Raw reads (million)	33.49	32.15	31.56	33.49	33.14	32.80	33.51	30.66
GC%	44.31	44.21	44.69	45.32	44.28	44.48	45.00	45.03
Mapped reads (million)	27.54	27.12	25.51	25.87	27.9	27.54	27.10	24.78
% Mapped reads	82.23	84.35	80.83	77.25	84.19	83.96	80.87	80.82
Reads in genes (million)	23.20	23.75	22.34	22.36	24.25	24.24	23.72	21.7
% reads in genes	69.27	73.87	70.79	66.77	73.17	73.9	70.78	70.78
Total genes >1 rpkm^	46045	46263	48202	48532	47014	43868	47795	47267
Genes >1 rpkm^ (%)	75.16	75.52	78.68	79.22	76.74	71.61	78.02	77.15
Genes >0 Reads	47097	47319	49253	49600	48099	45007	48858	48390
Total genes >0 Reads (%)	76.88	77.24	80.40	80.96	78.51	73.47	79.75	78.99

**Fig. 4:**
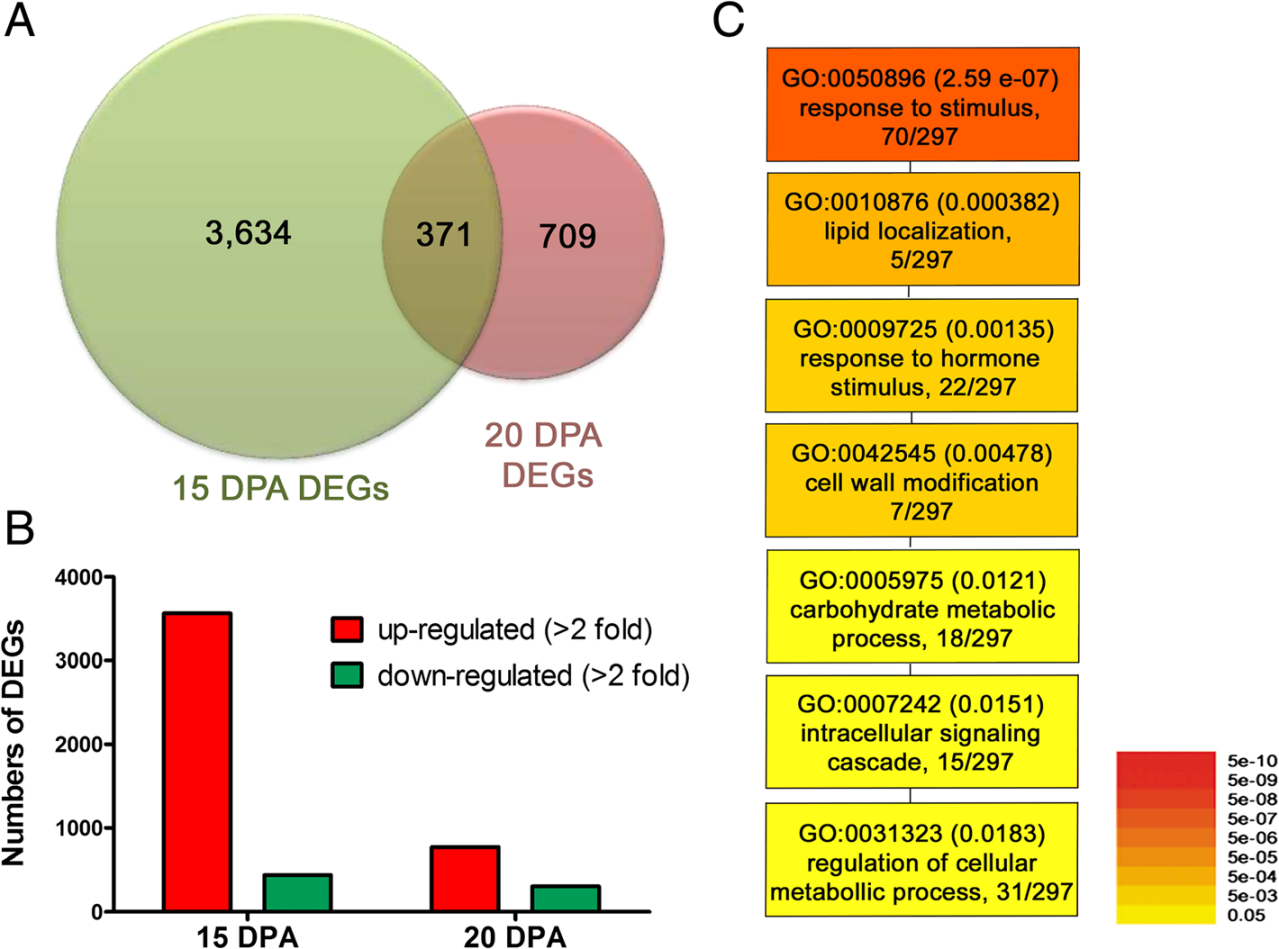
Summary of RNA-seq analysis comparing MD52ne and MD90ne. **a** Venn diagrams representing differentially expressed genes (DEGs) at two developmental stages (15 and 20 DPA) in MD52ne and MD90ne fibers. **b** Comparisons of up- or down-regulated DEGs at 15 and 20 DPA in MD52ne fibers. **c** GO analysis. Singular enrichment analysis was used to identify GO categories that were commonly found at both 15 and 20 DPA developing fibers from MD52ne. The color and numbers adjacent to the GO identifier represent *p*-values

Expressed mapped genes were further evaluated between two NILs using RPKM values for the respective time points. The false discovery rate (FDR) was set at 5 % to determine the threshold of *p* value in multiple tests and analyses. To judge the significance of gene expression difference, adjusted *p* ≤ 0.05 and the absolute value of log_2_ ratio ≥ 1 was used as the threshold [27]. Of the 37,675 expressed genes, 4005 and 1080 unique genes were significantly differentially expressed at 15 and 20 DPA, respectively between the NILs (Fig. 4a, Additional files 2 and 3). Of the 4005 differentially expressed genes (DEGs), 3565 and 440 unique genes were expressed as up- and down-regulated in MD52ne at 15 DPA respectively, while out of 1080 genes, 774 and 306 DEGs were expressed up- and down-regulated in MD52ne at 20 DPA, respectively (Fig. 4b, Additional files 4, 5, 6 and 7).

### Annotation and gene ontology analyses of DEGs in MD52ne fibers

GO enrichment analysis of the annotated DEGs by agriGo [28] showed that the DEGs involved in responses to stimuli and phytohormones, intracellular signaling, cellular metabolic process, cell wall modification, lipid localization, and carbohydrate metabolic process were commonly identified in developing MD52ne fibers at 15 and 20 DPA (Fig. 4c and Table 5). Numerous transcription factors regulated by growth stimulating phytohormones such as auxin, ethylene, and gibberellins (GA) were differentially expressed in developing MD52ne fibers (Table 5). Among them, 1-aminocyclopropane-1-carboxylic acid synthase 6 (ACC6) and 1-aminocyclopropane-1-carboxylate oxidase 4 (ACO4), key enzymes for synthesizing ethylene stimulating fiber elongation [29, 30], and *EIN3-binding F box protein 1* (*EBF1*) involved in ethylene signaling [31] were up-regulated at 15 and 20 DPA. *GAST1* encoding for a GA promoting growth enzyme [32] was highly up-regulated in MD52ne. We also conducted GO enrichment analysis using DEGs at 15 and 20 DPA separately (Additional file 8).

**Table 5:** Annotation of genes differentially expressed in MD52ne at 15 and 20 DPA fiber

Gene ID	Tair 10 ID	Description	Fold (MD52ne/MD90ne)	
			15 DPA	20 DPA
Response to hormone stimulus				
Gh_A08G0989	AT4G11280	1-aminocyclopropane-1-carboxylic acid synthase 6 (ACC6)	6.87	2.40
Gh_A07G0774	AT1G05010	1-aminocyclopropane-1-carboxylate oxidase 4 (ACO4)	6.35	4.28
Gh_A05G0085	AT2G25490	EIN3-binding F box protein 1 (EBF1)	5.01	2.28
Gh_A09G1760	AT4G17500	Ethylene responsive element binding factor 1	2.45	2.60
Gh_A12G1554	AT1G13260	Related to ABI3/VP1 1	4.21	4.76
Gh_D11G0427	AT5G07580	Integrase-type DNA-binding superfamily protein	7.92	2.64
Gh_A11G2242	AT5G43700	AUX/IAA transcriptional regulator family protein	10.19	7.99
Gh_A11G0443	AT2G14960	Auxin-responsive GH3 family protein	2.90	0.26
Gh_A08G1509	AT4G16780	Homeobox protein 2	5.01	2.40
Gh_D08G2557	AT5G57050	Protein phosphatase 2C family protein	3.56	2.12
Gh_D06G0657	AT4G26080	Protein phosphatase 2C family protein	2.68	2.24
Gh_D09G0145	AT5G15230	GAST1 protein homolog 4	3.96	3.19
Gh_A06G0128	AT5G25900	GA requiring 3	0.42	0.44
Gh_D06G0417	AT1G01720	NAC transcriptional regulator	2.72	3.23
Gh_D01G0514	AT4G27410	NAC transcriptional regulator	2.58	2.09
Inter & intra-cellular singaling cascade				
Gh_D08G0203	AT5G48940	Leucine rich repeat receptor-like kinase (LRR RLK)	376.83	701.62
Gh_D12G0208	AT1G49740	PLC-like phosphodiesterases superfamily protein	38.86	6.53
Gh_A11G1809	AT1G13680	PLC-like phosphodiesterases superfamily protein	5.13	4.88
Gh_D11G0427	AT5G07580	Integrase-type DNA-binding superfamily protein	7.92	2.64
Gh_A05G2005	AT5G12480	Calmodulin-domain protein kinase 7	12.98	15.75
Gh_A07G0928	AT5G59010	Protein kinase protein with tetratricopeptide repeat domain	4.75	2.48
Gh_A02G1701	AT2G17220	Protein kinase superfamily protein	3.77	2.06
Gh_A03G0393	AT5G59010	Protein kinase protein with tetratricopeptide repeat	3.11	2.71
Gh_D10G0338	AT2G33170	Leucine-rich repeat receptor-like kinase	2.18	2.33
Gh_D05G1232	AT1G11300	Protein serine/threonine kinases	0.41	2.94
Gh_D12G0893	AT1G54610	Protein kinase superfamily protein	0.39	0.18
Gh_D01G1037	AT4G20140	Leucine-rich repeat transmembrane kinase	0.34	0.30
Gh_A12G0190	AT1G49820	S-methyl-5-thioribose kinase	0.20	0.27
Gh_A03G0234	AT2G30500	Kinase interacting (KIP1-like) family protein	0.00	0.01
Gh_D12G2621	AT3G22370	Alternative oxidase 1A	0.03	0.17
Cell wall modification				
Gh_D13G0786	AT1G26770	Expansin A10	3.72	2.14
Gh_A04G0707	AT2G39700	Expansin A4	3.67	4.21
Gh_A05G1180	AT3G14310	Pectin methylesterase 3 (PME3)	3.41	6.70
Gh_A03G1432	AT4G03210	Xyloglucan endotransglucosylase/hydrolase 9 (XET9)	2.56	1.03
Gh_A11G0768	AT4G28850	Xyloglucan endotransglucosylase/hydrolase 9 (XET26)	3.97	1.34
Gh_D08G1309	AT4G02280	Sucrose synthase 3 (Sus3)	4.70	1.00
Gh_A07G0665	AT5G37180	Sucrose synthase 5 (Sus5)	13.97	0.94
Gh_D12G0298	AT3G29810	COBRA-like protein 2 precursor	1.23	11.09
Gh_A06G0439	AT3G12500	Chitinase/ Chitinase-like (CHI/CTL)	1.00	16.63
Gh_D06G0479	AT3G12500	Chitinase/ Chitinase-like (CHI/CTL)	1.84	10.61
Lipid localization				
Gh_A11G0232	AT2G45180	Lipid-transfer protein	27.06	100.50
Gh_D07G1618	AT5G48485	Lipid-transfer protein	3.54	2.78
Gh_A05G0073	AT2G10940	Lipid-transfer protein	4.66	4.32
Gh_D13G1979	AT4G30950	Fatty acid desaturase 6	2.42	2.44
Transcriptional regulation of cellular metabolic process				
Gh_D06G1433	AT1G29160	Dof-type zinc finger DNA-binding protein	21.26	3.01
Gh_A13G1525	AT1G22490	bHLH DNA-binding protein	10.53	7.80
Gh_D08G0923	AT5G57660	CONSTANS-like 5	4.64	2.46
Gh_D03G1189	AT1G07530	SCARECROW-like 14	2.65	2.71
Gh_A09G0414	AT5G52510	SCARECROW-like 8	2.07	2.22
Gh_D06G1966	AT1G80840	WRKY DNA-binding protein 40	5.40	3.25
Gh_D12G1243	AT1G13960	WRKY DNA-binding protein 4	2.35	3.28
Gh_D09G1456	AT5G56270	WRKY DNA-binding protein 2	2.02	2.29
Gh_A12G1763	AT1G57820	Zinc finger (C3HC4-type RING finger) protein	4.32	6.50
Gh_A01G1274	AT5G50570	Squamosa promoter-binding transcription factor	4.67	3.05
Gh_A11G2041	AT1G69560	MYB protein 105	3.66	2.51
Gh_A12G1244	AT4G21440	MYB-like 102	0.21	0.46
Gh_A04G0781	AT2G38250	Homeodomain-like superfamily protein	3.67	2.29
Gh_A08G1509	AT4G16780	Homeobox protein 2	5.01	2.40
Gh_A06G0833	AT4G32880	Homeobox gene 8	2.57	2.12
Gh_D08G2178	AT1G09250	Basic helix-loop-helix (bHLH) DNA-binding protein	5.23	2.38
Gh_D12G2057	AT5G46910	Transcription factor jumonji (jmj) zinc finger (C5HC2 type)	2.18	2.11
Gh_D11G0427	AT5G07580	Integrase-type DNA-binding superfamily protein	7.92	2.64
Gh_A10G0516	AT3G13890	MYB protein 26	9.28	0.87
Gh_A09G1074	AT5G12870	MYB protein 46	7.81	1.03

**Fig. 5:**
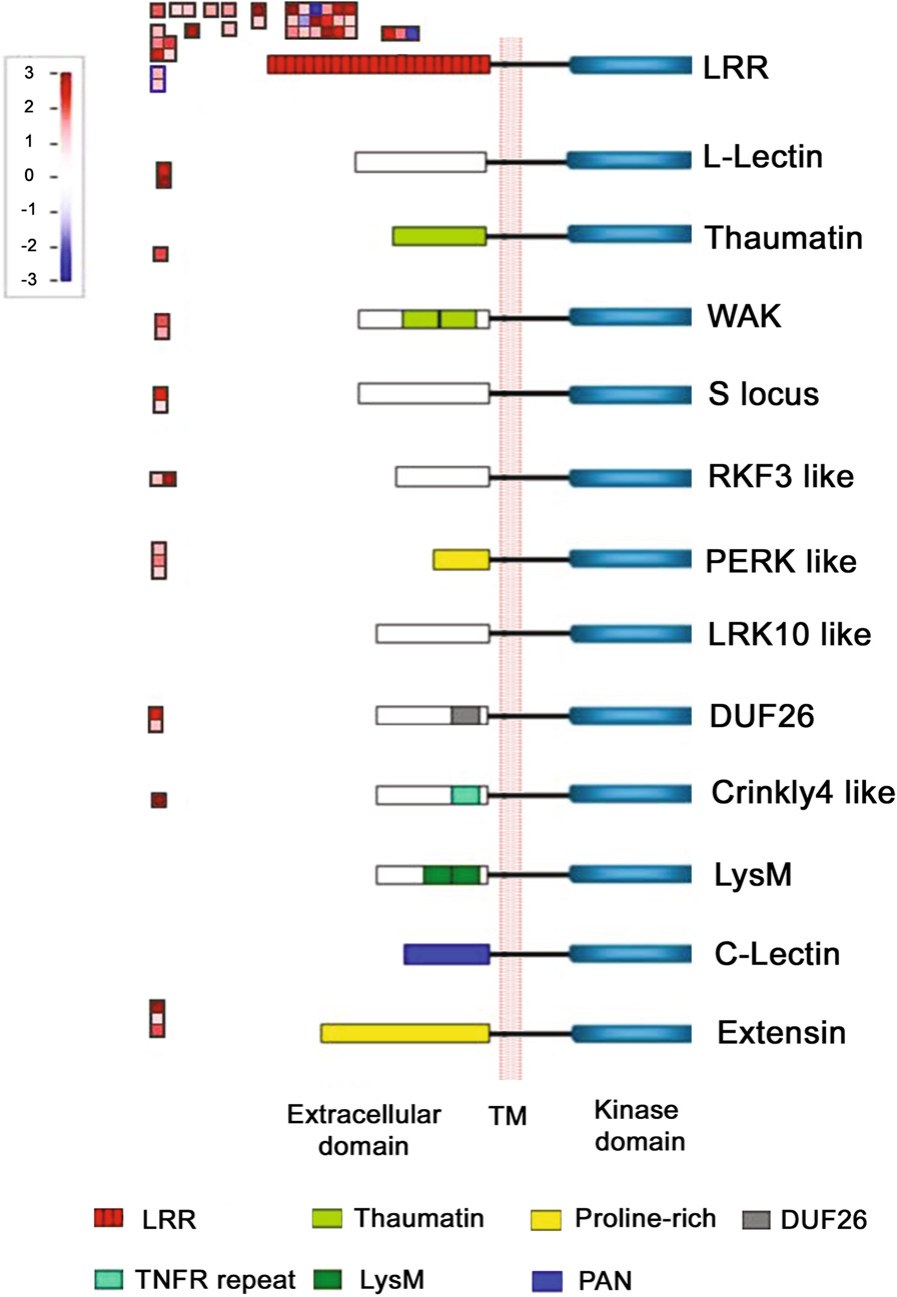
Differential expressions of *receptor-like kinases* (*RLK*s) in developing MD52ne fibers at 15 DPA. Differential expressions of RLKs in different classes were generated by MapMan. Red and purple represent up- and down- regulations, respectively. The RLKs contain three domains, including extracellular domain, transmembrane (TM), and kinase domain in a cytoplasmic side. C-lectin, RLKs with lectin-like motifs; Crinkly4-like, RLKs with crinkly4-like domains; DUF26, domain of unknown function 26; Extensin, RLK with extensin motif; L-lectin, RLKs with lectin-binding domains; LRK 10-like, RLK gene on Lr10 locus; LRR, leucine-rich repeats; LysM, RLKs with lysine motif; PERK-like, proline-rich extensin-like kinase; RKF3-like, receptor-like kinase in flowers 3; S-locus, RLK with S-domain; Thaumatin, RLK-like thaumatin protein; WAK, wall-associated kinase

Interestingly, multiple *receptor-like kinases* (*RLKs*) localized in plasma membranes were differentially expressed in the developing fibers of MD52ne as compared with MD90ne. Among the various classes of RLKs described in Fig. 5, the leucine rich repeat (LRR) RLKs containing three domains (LRR ligand binding motif, a transmembrane region and a kinase domain) were most frequently identified. The LRR RLKs have been recently suggested as a novel signaling pathway regulating plant cell wall integrity maintenance [33] and cellulose deposition in Arabidopsis [34, 35]. An *LRR RLK* (Gh_D08G0203) was one of the DEGs that were most highly up-regulated in developing MD52ne fibers at both 15 DPA (377 fold) and 20 DPA (702 fold) (Additional file 9).

Cotton genes encoding expansins [36, 37], pectin methylesterase (PME) [38], and xyloglucan endotransglucosylase/hydrolases (XET) [39] required for expanding the PCW during the fiber elongation stage were up-regulated in developing MD52ne fibers. Five *expansins* (Gh_D13G0786, Gh_A11G2917, Gh_A04G0707, Gh_A12G1619, and Gh_A12G1619), three *PME*s (Gh_A05G1180, Gh_D06G0865, and Gh_D07G0145), and five *XET*s (Gh_A03G1432, Gh_D02G1891, Gh_D05G0764, Gh_A11G0768, and Gh_D13G0290) were highly expressed in MD52ne fibers (Table 5). Four *Sus* (Gh_A07G0665, Gh_A08G1031, Gh_D08G1309, and Gh_D11G0438) and multiple lipid transfer proteins involved in fiber development [29, 40, 41] were co-expressed at actively elongating MD52ne fibers.

*COBRA-like protein 2,* whose sequence is similar to Arabidopsis *COBRA-like proteins* involved in cellulose microfibrils orientation in Arabidopsis [42], and *Chitinase*/*Chitinase-like* (*CHI/CTL*), similar to an Arabidopsis *CTL2* responsible for crystalline cellulose content in Arabidopsis [43], were specifically up-regulated in the SCW stage (20 DPA) of MD52ne. Several transcription factors including *NAC* (*No Apical Meristem*), *bHLH* (*basic helix-loop-helix*), *COL5* (*CONSTANS-like 5*), *WRKY*, and *zinc finger family protein* were differentially expressed in developing MD52ne fibers at both 15 and 20 DPA, whereas *MYB 26* and *46 transcription factors* orthologous to Arabidopsis *MYB26* (AT3G13890) and *46* (AT5G12870) involved in SCW biosynthesis [44, 45] were specifically up-regulated in developing MD52ne fibers at 15 DPA (Table 5).

### Validation of DEGs in MD52ne fibers

**Fig. 6:**
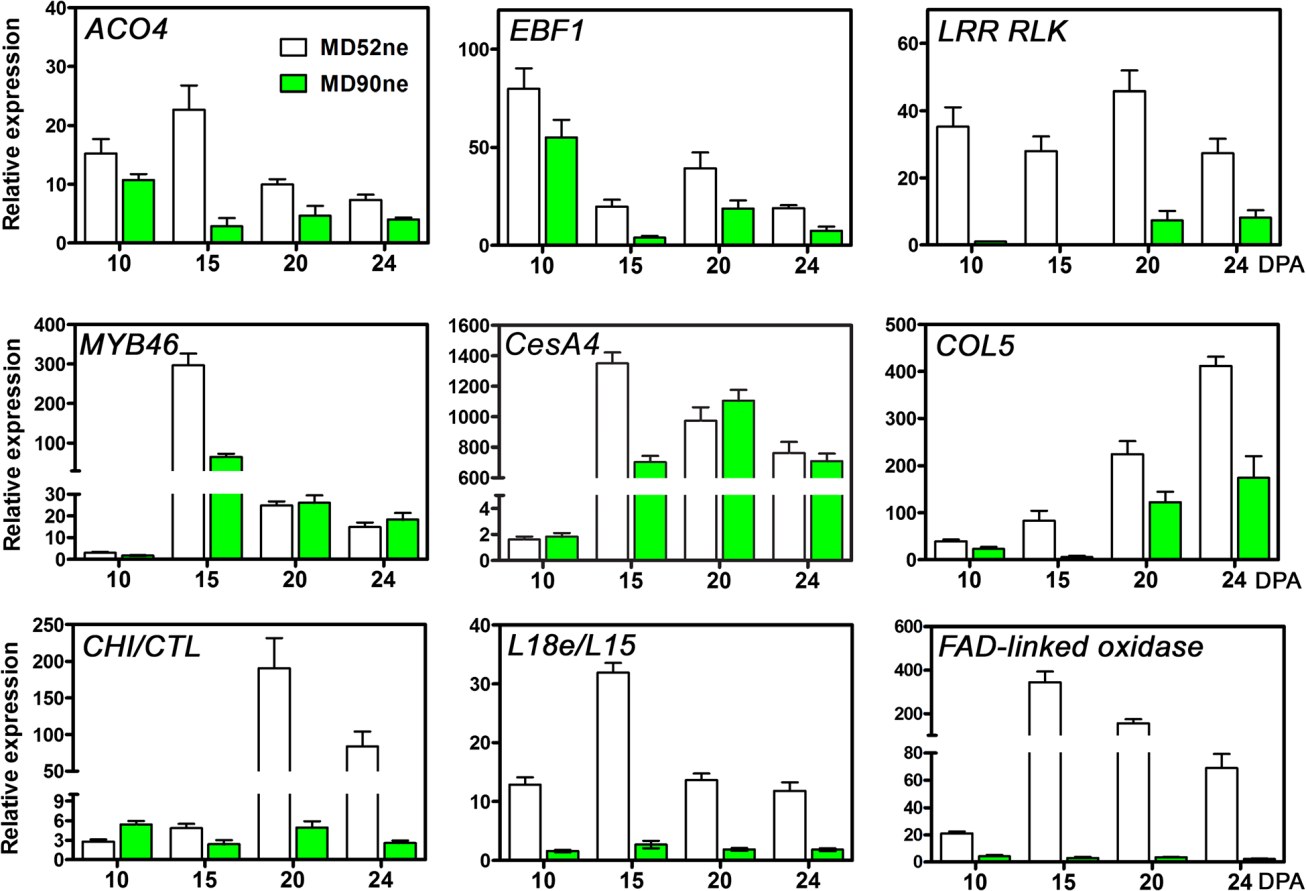
RT-qPCR validation of selected genes related to cell wall activity during fiber development. Three biological replications and three technical replications at four fiber development stages (10, 15, and 24 DPA) were used for RT-qPCR analyses. *ACO4, 1-aminocyclopropane-1-carboxylate oxidase 4,* or *ethylene forming enzyme* (Gh_A07G0774); *EBF1*, *EIN3-binding F box protein 1* (Gh_A05G0085); *LRR RLK*, *Leucine rich repeat receptor-like kinase* (Gh_D08G0203); *MBY46 transcription factor* (Gh_A09G1074); *CesA4, Cellulose synthase catalytic subunit A4* (Gh_A08G0421); *COL5*,*CONSTANS like 5* (Gh_D08G0923); *CHI/CTL*, *Basic-Chitinase/Chitinase-like* (Gh_D06G0439); *L18e/L15, Ribosomal L18e/L15 protein* (Gh_D02G1619); *FAD-linked oxidase* (Gh_D02G1214)

The expression patterns of the DEGs identified from RNA-seq data of the two NILs were validated with real time quantitative PCR (RT-qPCR) analysis. Based on GO enrichment analysis, 32 DEGs showing different expression pattern in the RNA-seq were selected for RT-qPCR using RNAs from developing fibers at 10, 15, 20 and 24 DPA. Comparative transcript levels of the all 32 DEGs obtained by both RT-qPCR and RNA-seq were presented in Additional file 10, and the RT-qPCR results from the 9 critical DEGs were shown in Fig. 6. The identical expression patterns of all the tested DEGs between RT-qPCR and RNA-seq indicated the reliability of both sequencing and the DEG filtering process.

*ACO4* and *EBF1*, which are both involved in the ethylene signaling pathway [29, 30], were expressed more in MD52ne than in MD90ne at all stages in PCW biosynthesis (10 DPA), transition (15 DPA), and SCW biosynthesis (20 and 24 DPA) (Fig. 6). The most significant up-regulation in all developmental stages was found in the *LRR RLK* that is a new signaling pathway of cell wall integrity [33]. A *transcription factor* showing sequence similarity to Arabidopsis *MYB 46* regulating biosynthesis of cellulose, hemicelluloses, and lignin [44, 46] was expressed more abundantly in MD52ne than in MD90ne specifically at the transition stage (15 DPA) between the PCW and SCW biosyntheses (Fig. 6). Consistently, c*ellulose synthase catalytic subunit A4* (*CesA4*) which is involved in the SCW biosynthesis of cotton fiber [47] showed an identical expression pattern to the *MYB46 transcription factor*. The expression pattern of a zinc finger protein *COL5* involved in cellular metabolic process was also highly abundant in MD52ne at all tested DPAs. A *CHI/CHI* (Gh_D06G0479) responsible for crystalline cellulose content in other plants [43, 48] was highly up-regulated in MD52ne as compared to MD90ne. During the active SCW biosynthesis stage (20–24 DPA), the transcript levels of *CHI/CHI* were 30 fold higher in MD52ne fibers than in MD90ne fibers. An *FAD-linked oxidase* and a *ribosomal L18e/L15 protein* (*L18e/L15*) were highly up-regulated in MD52ne over MD90ne in all developmental stages.

### DEGs at the QTL regions for bundle strength and fiber length

**Fig. 7:**
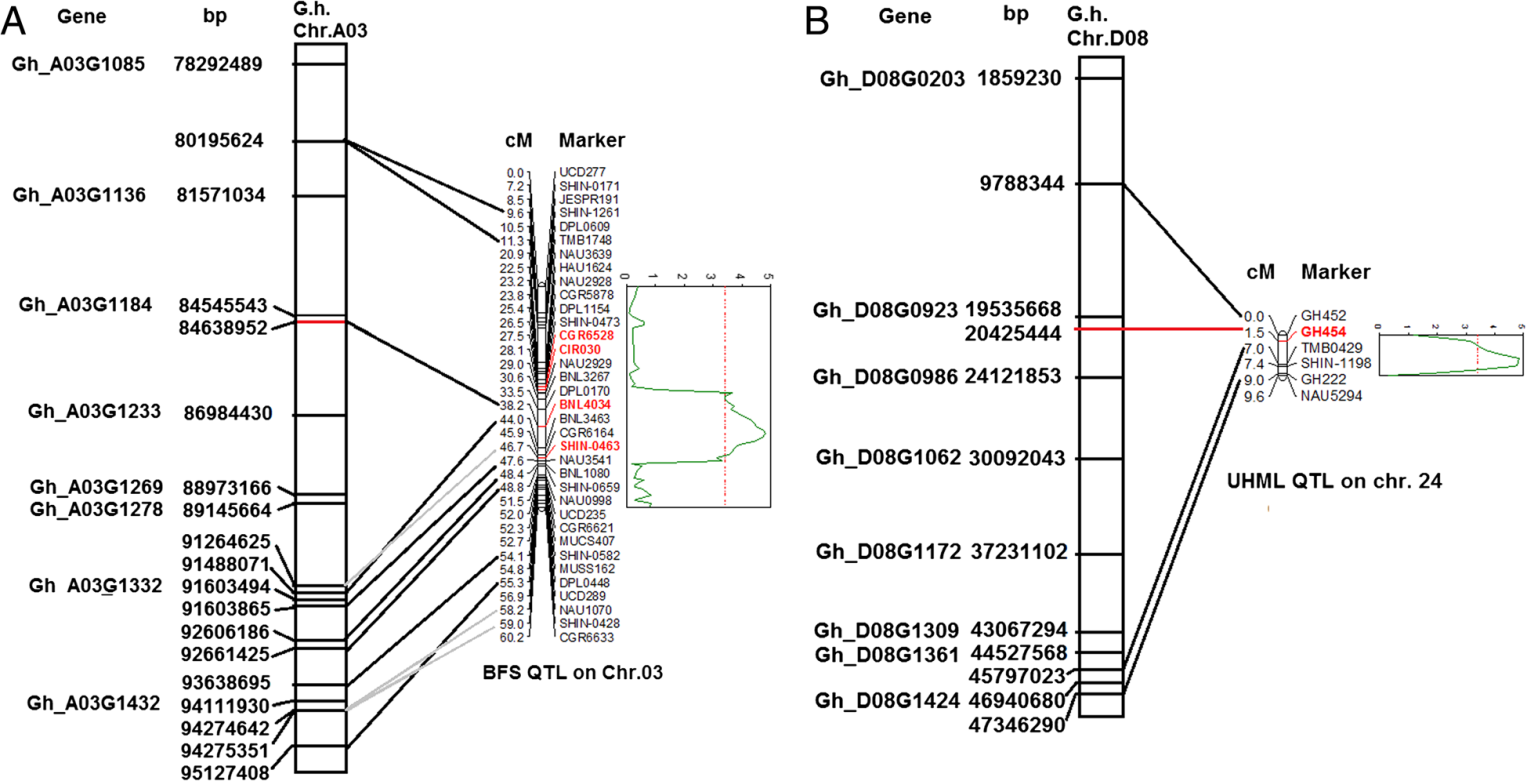
Alignments of DEGs in the physical map of *Gossypium hirsutum* TM-1 genome and the QTL regions related to bundle fiber strength (A) and UHML fiber length (B). Corresponding genes were highly abundant in MD52ne fibers than MD90ne. Genetic map locations [49] are shown in *centiMorgans* (cM) and physical locations are shown in *base pairs* (bp). Red markers were linked with the QTLs. **a** DEGs in A03 QTL. Gh_A03G1085 (*MADS-box transcription factor*), Gh_A03G1136 (*Response regulator 11*), Gh_A03G1184 and Gh_D02G1619 (*Ribosomal protein L18e/L15*), Gh_A03G1233 (*trehalose phosphatase/synthase 9*), Gh_A03G1269 (*LRR RLK*), Gh_A03G1278 (*membrane lipoprotein*), Gh_A03G1332 (*NAC 83*), Gh_A03G1432 (*XET 9*). **b** DEGs in D08 QTL. Gh_D08G0203 (*LRR RLK*), Gh_D08G0923 (*COL5*), Gh_D08G0986 (*LRR RLK*), Gh_D08G1062 (*Protein kinase*), Gh_D08G1172 (*NAC*), Gh_D08G1309 (*Sus 3*), Gh_D08G1361 (*Germin like protein 10*), Gh_D08G1424 (*WRKY*)

We previously identified stable quantitative trait loci (QTLs) associated with BFS and fiber length (UHML) using an F_2_ population derived from a cross between MD52ne and MD90ne [49]. The QTLs associated with BFS and UHML were linked with simple sequence repeat markers, BNL4034 and GH454 located on chromosome 3 (A03) and chromosome 24 (D08), respectively. We aligned the QTL regions along with the DEGs with the physical map of the *G. hirsutum* TM-1 genome [26, 50]. A total of 75 genes were differentially expressed either at 15 and 20 DPA in the QTL regions. Of the 75 DEGs, 17 genes (9 DEGs in the BFS QTL and 8 DEGs in the UHML QTL) up-regulated more than 2 fold in MD52ne were involved in cell wall modification based on GO analysis (Additional file 11). Three *LRR RLKs*, two *NAC transcription factors*, *COL5*, *MADS-box transcription factor*, and *WRKY transcription factor* involved in phytohormonal and RLK signaling pathways were located on the QTL regions (Fig. 7). *Trehalose-phosphatse / synthase 9, XET9, Sus3, and germin like protein* involved in cell wall biosynthesis or wall carbohydrate metabolisms were also found near the QTL regions. Two *ribosomal L18e/L15 proteins* were also located on chromosome A03 QTL location. Among them, the *ribosomal L18e/L15 protein* (Gh_D02G1619) at the A03 QTL and the *LRR RLK* (Gh_D08G0203) located near the D08 QTL were the DEGs that were mostly up-regulated at all developmental stages (Fig. 6).

## Discussion

### Greater force is required for breaking individual fibers of MD52ne than its NIL, MD90ne

The BFS value has been broadly used to evaluate fiber strength which is the breaking force of a fiber bundle. In addition to the breaking force, the BFS is also affected by variable fiber properties like length, fineness, maturity, and MIC involved in fiber-to-fiber interactions. The correlation analysis of fiber properties from the 384 F_2_ progenies from a cross between MD52ne and MD90ne showed that the BFS variation of the NILs was mainly determined by the breaking force with low effects from variable fiber properties related to fiber-to fiber interactions (Table 2). The fiber length showing 5–6 % differences between the NILs contributed a slight influence (12.9 %) on the BFS variances between the NILs, whereas the fiber thickness related properties having insignificant variations between the NILs had almost no effect on the BFS variances of the NILs (Tables 1 and 2). Since the BFS variance of the NILs is mainly driven by breaking force, the ratio of the bundle strength between the NILs was expected to be similar to the ratio of the individual fiber strength that is not affected by fiber-to fiber interactions. As predicted, the strengths of bundle (24 %) and individual fibers (22 %) from the MD52 were similarly higher than those from the MD90ne (Tables 1 and 3). As results, we concluded that the superior breaking force mainly contributed to the high bundle strength of the MD52ne fibers with a minor contribution from longer fiber. Compared with the cotton CSIL lines which have been used for studying fiber strength despite great potential effects from fiber length and thickness related properties due to their substantial variances among the CSILs [18, 19], the MD52ne and MD90ne are more ideal cotton NILs for dissecting molecular mechanisms of intrinsic fiber strength since their BFS variance was mainly affected by the breaking force but minimally regulated by other fiber properties involved in fiber-to fiber interactions within a fiber bundle.

Based on the fiber property analyses of the developing fibers whose crystallinity increased rapidly from 15 to 17 DPA (Fig. 3b), we determined that the transition from PCW to SCW biosynthesis stages began at approximately 15 DPA. Thus, we compared the transcript abundance in developing MD52ne and MD90ne fibers at two different time points: 15 DPA in which actively elongating fibers mainly consisted of PCW with low crystallinity (MD52ne, 18.1 %; MD90ne, 20.9 %), and 20 DPA in which SCW thickening fibers were composed of both PCW and SCW with high crystallinity (MD52ne, 38.4 %; MD90ne, 41.5 %). In the developing fibers at the transition stage, a new cell wall layer named as winding layer is deposited [51]. Based on the observation of high fiber strength in developing fibers (21 DPA) composed of a winding layer with minimum SCW [8], the winding layer has been speculated as a potential source of fiber strength [17, 52]. To determine if and how the winding layer contributes to the strength of bundle and individual fibers, further comprehensive studies may be necessary since single fiber strength of developing fibers was previously measured by an Instron tensile tester that was a prototype for measuring individual fiber strength [8] and caused high variability and inconsistency [53]. In our studies, high BFS values of the NILs were also detected at the transition stage (20 DPA) of the NILs (Fig. 3e). The BFS of developing MD52ne fibers (21.73 g/tex, 20 DPA) was significantly (*p* value, 0.0027) higher than that of developing MD90ne fibers (17.52 g/tex, 20 DPA).

### Ethylene and its networking phytohormonal pathways may be involved in superior fiber length development in MD52ne

**Fig. 8:**
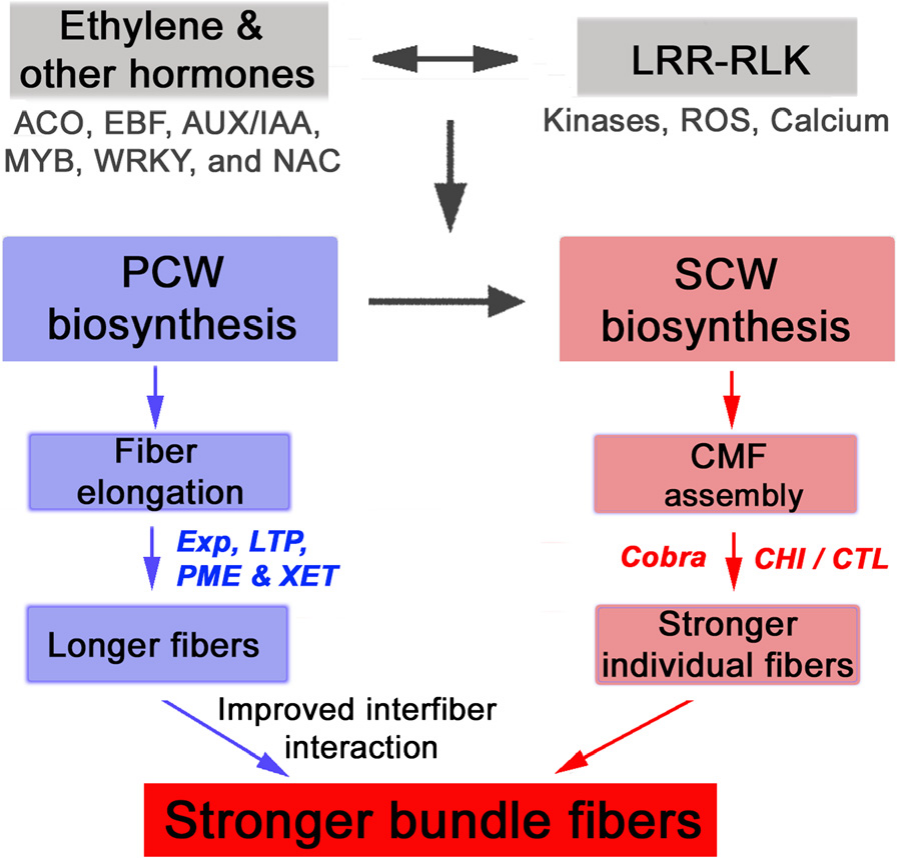
Proposed model for the mechanism responsible for high fiber bundle strength and individual fiber breaking force in MD52ne. Abbreviated names included in this model are: LRR RLK, Leucine rich repeat receptor-like kinase; PCW, primary cell wall; SCW, secondary cell wall; CME, cellulose micrfibrils; ACO, 1-aminocyclopropane-1-carboxylate oxidase; EBF, EIN3-binding F box protein; AUX/IAA, AUX/IAA transcription factor; MYB, MYB transcription factor: WRKY, WRKY transcription factor; NAC, NAC transcription factor; Exp, Expensin; LTP, Lipid transfer protein; PME, Pectin methylesterase; XET, Xyloglucan endotransglucosylase; Cobra, Cobra like protein; CHI/CTL, Chitinase/Chitinase-like protein; ROS, Reactive oxygen species

Comparative transcriptome analyses showed that transcripts related to ethylene and its networking auxin and GA signaling pathways for promoting fiber elongation were highly abundant in MD52ne (Table 5 and Fig. 4c). Ethylene gas is known as a major phytohormone stimulating fiber elongation [54]. Up-regulations of ethylene synthesizing genes like ACC and ACO as well as ethylene signaling gene like EBF are critical for active fiber elongation [29–31]. Consistent with the prior findings, *ACC6, ACO4, EBF1*, and *ERF1* involved in ethylene biosynthesis and signaling pathway required for fiber elongation were highly expressed in elongating MD52ne fibers whose length (UHML) was longer than its NIL, MD90ne (Table 5 and Fig. 6). In addition, transcripts (*AUX/IAA*, *auxin-responsive GH3 protein*, and *GAST1*) involved in auxin and GA were required for differentiating and elongating fibers [41, 55]. For promoting fiber elongation, multiple *expansins* involved in loosening the cell walls and *lipid transfer proteins* involved in fiber elongating [29] were also up-regulated in the MD52ne fibers (Table 5 and Fig. 8). A large set of genes (*XET, PME*, and *Sus*) involved in xyloglucan and pectin biosynthesis and carbohydrate metabolism requiring cotton fiber elongation [56] was also enriched in the elongating MD52ne fibers (Tables 5 and 6). A *XET* (Gh_A03G1432) and a *Sus* (Gh_D08G1309) were linked with the QTLs associated with BFS and UHML (Fig. 7).

### Receptor-like kinase signaling pathway regulating cellulose deposition and maintaining cell wall integrity may be involved in superior fiber strength development in MD52ne

In addition to phytohormone and transcriptional networks controlling plant growth and development, receptor-like kinases (RLKs) have been found as novel regulators for both plant development and stress responses [33, 57]. Among the various RLK classes described in Fig. 5, RLKs containing a leucine-rich repeat (LRR) were most frequently identified from developing MD52ne fibers. Three LRR RLKs were also found in the two QTLs associated with BFS and length (Table 5, Figures 6 and 7). In Arabidopsis elongating root tips and seeds, two LRR RLKs named FEI 1 and 2 have been reported to play a role in cellulose deposition in Arabidopsis elongating root tips [34] and seed coat [35]. ACC (1-aminocyclopropane-1-carboxylic acid) that is essential for ethylene signaling has been suggested to be a signaling molecule for FEI 1 and 2 [58], so both ethylene and LRR RLK signaling are most likely involved in cellulose deposition in elongating tissues (Fig. 8). Other LRR RLKs whose ligands are unknown are reported as a regulator of the SCW formation in Arabidopsis [59] and popular trees [60]. A cotton LRR RLK named GhRLK1 located in the plasma membrane was reported to be induced during active SCW synthesis stage [61]. Therefore, LRR RLK signaling pathways might be involved in mediating a coordination of cell elongation and SCW biosynthesis during cotton fiber development as suggested in other plants [57, 58].

### Temporal regulation of the genes involved in crystalline cellulose assembly at secondary wall biosynthesis stage of MD52ne fibers

Three genes (Gh_D12G0298, Gh_A13G0320, and Gh_D13G0359) encoding COBRA-like protein were specifically up-regulated during the SCW biosynthesis stage in MD52ne fibers (Table 5). *COBRA-like protein 2,* a member of the GPI-anchored COBRA-like family, has been recently identified to play a role in crystalline cellulose deposition in Arabidopsis seed coat [62]. When a COBRA-like protein was deficient in brittle culm1 rice mutant [63] or brittle stalk2 maize mutant [64], mechanical strength of the stems was reduced. A COBRA-like protein interacting with cellulose modulates cellulose assembly in rice [42]. Therefore, the three *COBRA-like proteins* up-regulated at the SCW stage of MD52ne fibers can be candidate genes that contribute to the superior strength of MD52ne (Fig. 8).

Four *CHI/CTL* genes (Gh_D06G0479, Gh_A06G0439, Gh_A10G1271, and Gh_D09G2016) specifically up-regulated in the SCW stage of MD52ne fibers were similar to the sequences of *Chitinase* (*CHI*) and *Chitinase like protein* (*CTL*) in other plants. *CTL2* is responsible for crystalline cellulose content in Arabidopsis [43], whereas *CHI* is a pathogenesis-related gene responding to biotic stress in rice and maize [65, 66]. Thus, the real function of *CHI/CTL* genes identified from MD52ne remains to be determined.

### Temporal regulation of genes at transition stage of MD52ne fibers

We previously reported temporal up-regulation of SCW biosynthesis-related genes at the transition from the PCW to SCW stage in the MD52ne fibers based on the transcriptome profiles performed with the first generation of cotton oligonucleotide microarray [16, 17]. The present transcriptome analyses performed with RNA-seq and *G. hirsutum* genome sequence identified many more DEGs in the MD52ne fibers (Additional file 12) than the previous microarray analysis [17]. The RNA-seq analysis also showed that some SCW biosynthesis-related genes such as *MYB26*, *MYB4*6, and *CesA4* [44, 45, 47] were up-regulated specifically at 15 DPA (Table 5 and Fig. 6). In contrast, other 26 *CesA*s identified by the RNA-seq did not show temporal up-regulation at the transition stage. For further analysis of the temporal regulation of SCW biosynthesis-related genes, we retrieved 181 Arabidopsis genes from PlaNet [67] that are temporally and spatially co-expressed during SCW biosynthesis in Arabidopsis. Among the MD52ne genes ortholgous to the 181 Arabidopsis SCW-related genes, 64 genes (35.4 %) showed temporal up-regulation in 15 DPA developing MD52 fibers (Additional file 13), whereas others showed no temporal up-regulation. In addition to the limited numbers of up-regulated SCW genes in the MD52ne over MD90ne, the identical levels of the crystalinity between the NILs (Fig. 2B) might imply that the genes related to SCW cellulose biosynthesis were less involved in the superior fiber strength of the MD52ne than the genes related to SCW cellulose assembly and wall integrity.

## Conclusions

The demand of high fiber strength has been increased dramatically with the advent of modern high speed spinning technology for producing yarn. Cotton researchers have tried to improve this trait in *G. hirsutum* genetic backgrounds. MD52ne was proven to have higher fiber strength than its NIL MD90ne. This study was conducted to unveil the molecular mechanisms behind the formation of superior individual fiber strength, which is correlated with yarn strength, in MD52ne using RNA-seq technology. The bundle strength of the MD52ne fibers predominantly depends on individual fiber strength combined with fiber length. Comparative transcriptome analyses of the NILs suggested that the superior strength of MD52ne fibers was potentially related to two signaling pathways (Fig. 8): one is ethylene and its interconnected phytohormonal pathways involved in fiber elongation and interfiber interactions, and the other is RLKs signaling pathways involved in regulating cell wall integrity and potentially mediating a coordination of cell elongation and SCW biosynthesis. Several secondary cell wall biogenesis related genes and transcription factors such as *COBRA-like protein*, *CHI/CTL*, *NAC, WRKY, COL5, Zinc finger family protein* and *MYB* were up-regulated in MD52ne developing fibers. The superior BFS of MD52ne fibers might be the result of high individual fiber strength with a minor contribution from longer fiber length. The longer fibers may increase the fiber-to-fiber interactions and are likely the result of differential regulation of some PCW related genes. The improved individual fiber strength of MD52ne is likely related to pathways regulating cell wall integrity.

## Methods

### Plant material

The cotton NILs MD52ne and MD90ne were bred and provided by Dr. William Meredith of USDA-ARS-SEA (Additional file 14) [14, 15]. The two NILs were grown at two different fields for two growing seasons and at a greenhouse for one growing season. To compare fiber physical properties between the two NILs and determine variations in each NIL plant, ten individual plants of each NIL were grown at a field located in Stoneville, MS in 2012. The soil type in Stoneville is Bosket fine sandy loam. The mature fibers were collected from each plant of each NIL. To perform comparative transcriptome analyses, three biological replications (approximately 40 cotton bolls per replication) at each developmental time point (10, 13, 15, 17, 20, 24, 28, 33, 37, 44, and 48 DPA) were collected from 50 plants of the two NILs grown at a field located in New Orleans, LA in 2013. The soil type in New Orleans was Aquent dredged over alluvium in an elevated location to provide adequate drainage. Fiber samples from 384 F_2_ progeny plants derived from crosses between MD90ne and MD52ne were collected at Stoneville, MS, USA in 2012 as described in Islam et al. [49]. All naturally-open bolls were manually harvested from each of the F_2_ plants. Developing fibers (10–37 DPA) were manually collected from ovules and mature fibers (44 DPA) were ginned using a laboratory roller gin. The collected fibers were frozen immediately with liquid nitrogen for RNA extraction or dried in 40 °C incubator for physical property analyses. To extract additional RNAs that were used for verification of the transcriptome results, three biological replications of cotton fibers at each developmental time point (10–44 DPA) were collected from the two NILs grown in 5 gal pots with Metro-Mix 360 in a greenhouse located in New Orleans, LA. Throughout all processes from planting, tagging, harvesting, and ginning, the two NILs grown side by side were equivalently treated.

### Fiber property measurements

For measurements of fiber properties from cotton fibers, fibers were pre-equilibrated with 65 ± 2 % humidity at 21 ± 1 °C for 48 h. All fiber properties were obtained from instruments that were properly calibrated according to the manufacturers’ instructions and standard cotton fibers were obtained from USDA-AMS.

BFS (g/tex), UHML (inch), FE (%), and MIC values of the mature fibers from the two NILs were obtained from five replicates measured by HVI (USTER Technologies Inc., Knoxville, TN). To determine BFS from developing fibers, a stelometer (SDL Atlas, Stockport, England) was used with three replicates of fiber samples. BFS is given as tenacity, expressed as kilonewton meters per kilogram, and is the force required to break a bundle of fibers of a specific gravimetric linear density. For measuring the breaking force of individual fibers, Favimat (Textechno, Mönchengladbach, Germany) was used with 303 individual fibers and a 13 mm length gauge, according to Delhom et al. [68].

AFIS maturity ratio and fineness were measured using Uster® AFIS-Pro (USTER Technologies Inc., Knoxville, TN). The average AFIS fiber data were obtained from five replicates with 5000 fibers per replicate.

For measuring the gravimetric fineness (mtex, mg km^−1^) of the fibers, three hundred fibers were combed, cut at the top and bottom to leave them 15 mm long, and measured by a microbalance [69]. Average gravimetric fineness was calculated from the three measurements.

Circularity (θ) representing the degree of fiber cell wall thickness was directly measured from light microscopic images from cross-sectioned fibers [70]. Average cell wall area (A), excluding lumen and perimeter (P) of the fiber cross sections, was measured from 300 cross-sections using the image analysis software according to Xu and Huang [71]. The obtained circularities from the equation, θ = 4πA/P^2^ [72] were converted to maturity ratio (MR) using the equation, MR = θ / 0.577 [73].

### ATR-FTIR spectral collection and data analysis

All spectra were collected with an FTS 3000MX FTIR spectrometer (Varian Instruments, Randolph, MA) equipped with a ceramic source, KBr beam splitter, and deuterated triglycine sulfate (DTGS) detector. The ATR sampling device utilized a DuraSamplIR single-pass diamond-coated internal reflection accessory (Smiths Detection, Danbury, CT), and a consistent contact pressure was applied by way of a stainless steel rod and an electronic load display. At least six measurements at different locations for individual samples were collected over the range of 4000–600 cm^−1^ at 4 cm^−1^ and 16 co-added scans. All spectra were given in absorbance units and no ATR correction was applied. Following the import to GRAMS IQ application in Grams/AI (Version 9.1, Thermo Fisher Scientific, Waltham, MA), the spectra were smoothed with a Savitzky–Golay function (polynomial = 2 and points = 11). Then, the spectral set was loaded into Microsoft Excel 2000 to assess cotton crystallinity and maturity by using a previously proposed algorithm analysis [23, 24]. In the original concept of assessing cellulose maturity (M_IR_) and crystallinity index (CI_IR_) from IR measurement [22–24], the key wavelengths was identified and then two algorithms (R_1_ and R_2_) were developed to estimate the degree of cotton cellulose M_IR_ and CI_IR_ by representing the R_1_ and R_2_ values. Both mean value and standard deviation for each fiber sample were used for the comparison between two types of varieties.

### RNA extraction, library preparation and sequencing

Total RNA was extracted from the developing cotton fibers (10, 15, 20 and 24 DPA) using the Sigma Spectrum™ Plant Total RNA Kit (Sigma-Aldrich, St. Louis, MO) with DNase1 digestion according to the manufacturer’s protocol. The quality and quantity of total RNA were determined using a NanoDrop 2000 spectrophotometer (NanoDrop Technologies Inc., Wilmington, DE) and an Agilent Bioanalyzer 2100 (Agilent Technologies Inc., Santa Clara, CA). The RNA samples of two biological replications at two different developmental stages (15 and 20 DPA) from both NIL fibers were sent to Data2Bio LLC (Ames, Iowa) for library preparation and subsequent paired-end Illumina mRNA sequencing according to the methods that were previously described [74].

### RNA-seq data processing

The raw RNA-seq reads were trimmed with SICKLE (https://github.com/najoshi/sickle) using a quality score cutoff of 20. Then, the RNA-seq reads were aligned to the *Gossypium hirsutum* draft genome [26] with the GSNAP software program [75]. Reads mapped to each annotated gene were counted with Bedtools software [76].

### Identification of differentially expressed genes

Differential gene expression was calculated by the negative binomial method of the EdgeR software using the tagwise estimation of dispersion [77]. RPKM was used to estimate gene expression levels as calculated: RPKM = [10^9^/*NL*] C, where C stands for the number of reads that could map to the target unigene, N represents the number of reads that could map to at least one unigene, and L refers to the length of the target unigene. The accuracy of the test result was corrected by FDR. In this study, FDR < 0.05 and the absolute value of the log_2_ ratio (in which ‘ratio’ refers to the fold change in expression of the target unigene in among libraries) were used to select DEGs.

### Validation of RNA-seq results with RT-qPCR

The experimental procedures and data analysis related to RT-qPCR were performed according to the Minimum Information for Publication of Quantitative Real-Time PCR Experiments guidelines [70]. Four fiber development stages (10, 15, 20 and 24 DPA) were used for RT-qPCR analyses for validating the RNA-seq result of selected genes. The detailed description of cDNA preparation, qPCR, and calculations were previously reported [20]. Specific primer pairs were designed from 32 DEGs for validation of the RNA-seq. The endogenous reference genes used in the RT-qPCR reactions were the 18S rRNA (U42827), and α-tubulin 4 (AF106570). The reference and target gene primer sequences are shown in Additional file 15. Three biological replications and three technical replications for each time-point were used for RT-qPCR.

### Gene annotation analyses

RNA-seq data obtained from 15 and 20 DPA fibers of two NILs were first subjected to Venn analysis utilizing BioVenn [78] to determine which DEGs were common between two time-points. To assist in the identification of biological processes represented in the data, GO enrichment analysis was performed using the agriGO Singular Enrichment Analysis [28]. The statistical test method used was the Fisher’s exact test (significance level 0.05). For metabolic analysis, MapMan software [79] was used to identify and illustrate metabolic overview of cell wall related molecules.

### Ethics approval and consent to participate

Not applicable.

### Availability of supporting data

All supporting data can be found within the manuscript and its additional files. The biological sequences were deposited in the Sequence Read Archive (SRA), National Center for Biotechnology Information (NCBI) under the accession numbers SRS843151, SRS843159, SRS843160, and SRS843163.

## Additional files

Additional file 1:
**Name of mapped gene in** ***Gossypium hirsutum*** **(TM-1) and their annotation with Arabidopsis homeolog genes.** This table contains 61,263 allotetraploid cotton genes that aligned to *Gossypium hirsutum* (TM-1) draft genome. Mapped genes were also annotated with Arabidopsis homeolog genes and provide their description. The physical locations including chromosome name of each gene are also provided. First column have *G. hirsutum* (TM-1) gene name followed by Arabidopsis gene name, TAIR 10 gene description, *G. hirsutum* chromosome number, physical location of the gene and types of strand (+/-). (XLSX 3358 kb) Additional file 2:
**Differentially expressed genes at 15 DPA between MD52ne and MD90ne.** This table contains 4005 allotetraploid cotton genes that differentially significantly express between MD52ne and MD90ne in 15 DPA developing fibers. The threshold absolute value of log_2_ ratio was ≥ 1 and *p* value ≤ 0.05. Comparison of expression level between MD52ne and MD90ne is also included as fold change. First column have *G. hirsutum* (TM-1) gene name followed by Arabidopsis gene name, TAIR 10 gene description, log_2_ ratio of reads per kilo-base per million reads (RPKM) between MD52ne and MD90ne, corresponded *p* value, adjusted *p* value and fold change (MD52ne over MD90ne). (XLSX 428 kb) Additional file 3:
**Differentially expressed genes at 20 DPA between MD52ne and MD90ne.** This table contains 1080 allotetraploid cotton genes that differentially significantly express between MD52ne and MD90ne in 20 DPA developing fibers. The threshold absolute value of log_2_ ratio was ≥ 1 and *p* value ≤ 0.05. Comparison of expression level between MD52ne and MD90ne is also included as fold change. First column have *G. hirsutum* (TM-1) gene name followed by Arabidopsis gene name, TAIR 10 gene description, log_2_ ratio of reads per kilo-base per million reads (RPKM) between MD52ne and MD90ne, corresponded *p* value, adjusted *p* value and fold change (MD52ne over MD90ne). (XLSX 127 kb) Additional file 4:
**Differentially expressed up-regulated genes at 15 DPA in MD52ne.** Three thousand five hundred sixty five differentially expressed up-regulated genes at 15 DPA in MD52ne are included in this table. First column have *G. hirsutum* (TM-1) gene name followed by Arabidopsis gene name, TAIR 10 gene description, log_2_ ratio of reads per kilo-base per million reads (RPKM) between MD52ne and MD90ne, corresponded *p* value, adjusted *p* value and fold change (MD52ne over MD90ne). (XLSX 371 kb) Additional file 5:
**Differentially expressed down-regulated genes at 15 DPA in MD52ne.** Four hundred forty differentially expressed up-regulated genes at 15 DPA in MD90ne are included in this table. First column have *G. hirsutum* (TM-1) gene name followed by Arabidopsis gene name, TAIR 10 gene description, log_2_ ratio of reads per kilo-base per million reads (RPKM) between MD52ne and MD90ne, corresponded *p* value, adjusted *p* value and fold change (MD52ne over MD90ne). (XLSX 63 kb) Additional file 6:
**Differentially expressed up-regulated genes at 20 DPA in MD52ne.** Seven hundred seventy four differentially expressed up-regulated genes at 20 DPA in MD52ne are included in this table. First column have *G. hirsutum* (TM-1) gene name followed by Arabidopsis gene name, TAIR 10 gene description, log_2_ ratio of reads per kilo-base per million reads (RPKM) between MD52ne and MD90ne, corresponded *p* value, adjusted *p* value and fold change (MD52ne over MD90ne). (XLSX 91 kb) Additional file 7:
**Differentially expressed down-regulated genes at 20 DPA in MD52ne.** Three hundred six differentially expressed up-regulated genes at 15 DPA in MD90ne are included in this table. First column have *G. hirsutum* (TM-1) gene name followed by Arabidopsis gene name, TAIR 10 gene description, log_2_ ratio of reads per kilo-base per million reads (RPKM) between MD52ne and MD90ne, corresponded *p* value, adjusted *p* value and fold change (MD52ne over MD90ne). (XLSX 42 kb) Additional file 8:
**GO analysis.** Singular enrichment analysis was used to identify GO categories that were differentially expressed at 15 (A) and 20 (B) DPA developing fibers from MD52ne. The color and numbers adjacent to the GO identifier represent *p*-values. This file contains the results of GO enrichment analysis using differentially expressed genes at 15 and 20 DPA separately. (DOCX 198 kb) Additional file 9:
**Differential expressions of** ***receptor like kinases*** **(** ***RLK*** **s) in developing MD52ne fibers at 20 DPA.** Differential expressions of RLKs in different classes were generated by MapMan. Red and purple color represent up and down regulated, respectively. The RLKs contain three domains including extracellular domain, transmembrane (TM), and kinase domain in a cytoplasmic side. C-lectin, RLKs with lectin-like motifs; Crinkly4-like, RLKs with crinkly4-like domains; DUF26, domain of unknown function 26; Extensin, RLK with extensin motif; L-lectin, RLKs with lectin-binding domains; LRK 10-like, RLK gene on Lr10 locus; LRR, leucine-rich repeats; LysM, RLKs with lysine motif; PERK-like, proline-rich extensin-like kinase; RKF3-like, receptor-like kinase in flowers 3; S-locus, RLK with S-domain; Thaumatin, RLK-like thaumatin protein; WAK, wall-associated kinase. This file contains the results of MapMan analysis of different RLKs using differentially expressed genes at 20 DPA. (DOCX 135 kb) Additional file 10:
**RT-qPCR validation of some selected differentially expressed genes from RNA-seq data during fiber development.** This file contains the results of qPCR analysis and RNA-seq expression data performed on 32 genes that were used to compare fold expression levels between MD52ne and MD90ne. Four (10, 15, 20 and 24 DPA) and two (15 and 20 DPA) developing fiber samples were used for RT-qPCR and RNA-seq, respectively. RT-qPCR values were corrected to Tubilin and 18S gene for each sample. For each treatment group three qPCR measurements were taken for each of three biological replicates and then averaged. (DOCX 644 kb) Additional file 11:
**Names of the important genes related to cotton fiber cell wall development are located near the identified QTL regions.** This table contains differentially expressed genes from RNA-seq data that physically located near the two previously reported QTLs associated with BFS and UHML located on chromosomes 03 and 24, respectively by Islam et al. 2014 [49]. (XLSX 12 kb) Additional file 12:
**Comparison of deferentially expressed genes between RNA-seq and microarray of Hinchliffe et al. 2010.** These figure compare the number of differential expressed genes between RNA-seq (RNA) results of this study and microarray (MA) data reported by Hinchliffe et al. 2010 [17]. RNA-seq and microarray data were generated from 15, 20 and 16, 20 DPA developing fiber samples. A) Total DE genes at both time point; B) DE only expressed in 15 DPA for RNA seq and 16 DPA for microarray data; C) DE only expressed in 20 DPA for both RNA seq and microarray data. (DOCX 320 kb) Additional file 13:
**Cotton MD52ne transcript levels compared with Arabidopsis genes that are co-expressed at secondary wall biogenesis.** This table contains 64 secondary cell wall (SCW) biosynthesis related genes retrieved from Arabidopsis genes from PlaNet [67] that are temporally and spatially co-expressed during SCW biosynthesis in Arabidopsis. Those 64 genes were up-regulated in MD52ne. (DOCX 20 kb) Additional file 14:
**Crossing scheme for developing upland cotton near isgenic lines MD52ne and MD90ne.** This figure contains crossing scheme for developing upland cotton near isogenic lines MD52ne and MD90ne has taken from Islam et al. 2014 [50]. In parentheses R is for recurrent and D is for donor parent for the respective cross. JCPC, DP, MD and FTA are the germplasm name and stand for John Cotton Poly Cross, Deltapine, Mississippi Delta and ARS strain from Pee Dee experiment station, Florescence, SC. (DOCX 131 kb) Additional file 15:
**Primer name and their sequences used in this study for RT-qPCR validation.** This file contains 32 RT-qPCR primer pair sequences that were used to validate RNAseq expression data. Corresponding gene name and descriptions are also included here. (XLSX 13 kb)

## Abbreviations

AFIS: Advanced fiber information system
CTL: Chitinase-like
DEG: Differentially expressed gene
DPA: Days post anthesis
FBS: Fiber bundle strength
FDR: False discovery rate
FE: Fiber elongation
FTIR: Fourier transform infrared spectroscopic
GF: Gravimetric fineness
HVI: Heavy Volume Instrument
GO: Gene ontology
MIC: Micronaire
NIL: Near-isogenic line
PCW: Primary cell wall
RPKM: Reads per kilobase per million reads
RT-qPCR: Reverse transcription quantitative polymerase chain reaction
SCW: Secondary cell wall
TF: Transcription factor
UHML: Upper-half mean length
