# Supplementary material for: Comparative fiber property and transcriptome analyses reveal key genes potentially related to high fiber strength in cotton (Gossypium hirsutum L.) line MD52ne
Source: BMC Plant Biol. 2016 Feb 1;16:36. doi: 10.1186/s12870-016-0727-2 (PMC4736178; doi:10.1186/s12870-016-0727-2)
Supplement: Additional file 8: — GO analysis. Singular enrichment analysis was used to identify GO categories that were differentially expressed at 15 (A) and 20 (B) DPA developing fibers from MD52ne. The color and numbers adjacent to the GO identifier represent p-values. This file contains the results of GO enrichment analysis using differentially expressed genes at 15 and 20 DPA separately. (DOCX 198 kb) [file 12870_2016_727_MOESM8_ESM.docx]

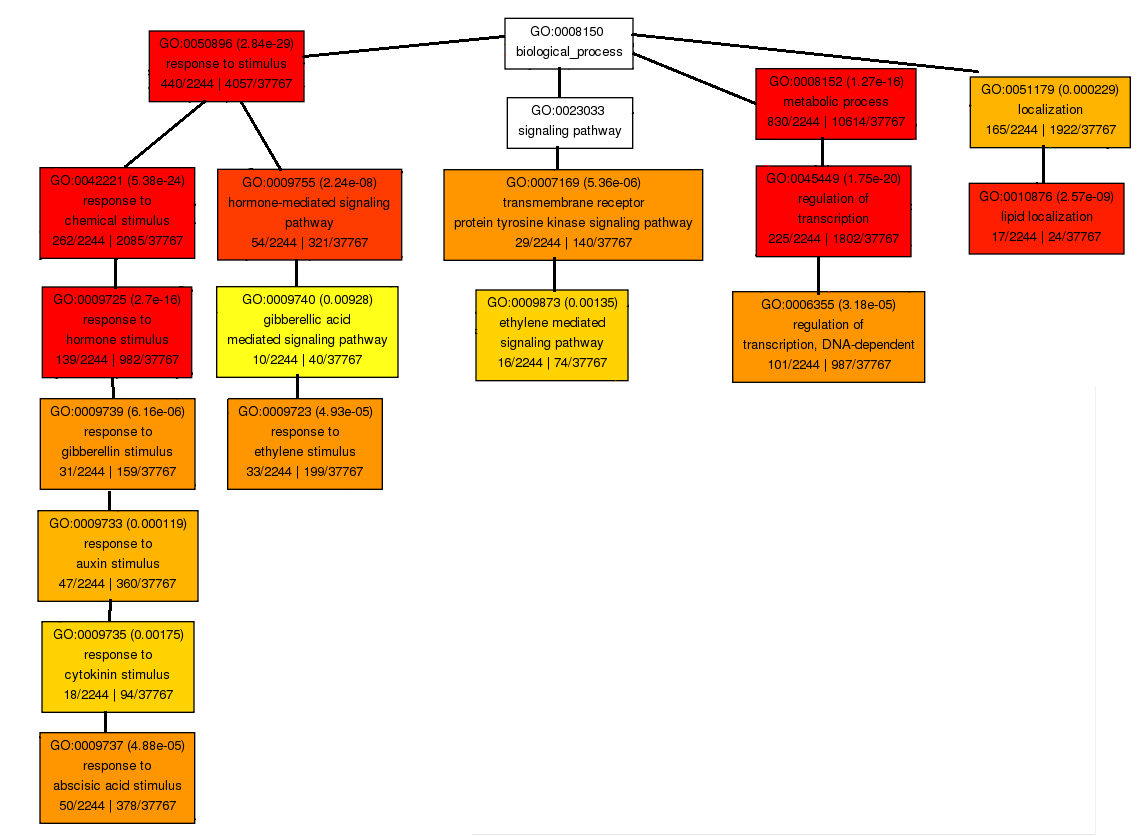
Additional file 8 .GO analysis. Singular enrichment analysis was used to identify GO categories that were differentially expressed at 15 (A) and 20 (B) DPA developing fibers from MD52ne. The color and numbers adjacent to the GO identifier represent *p*-values.

**A)**


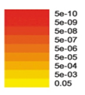


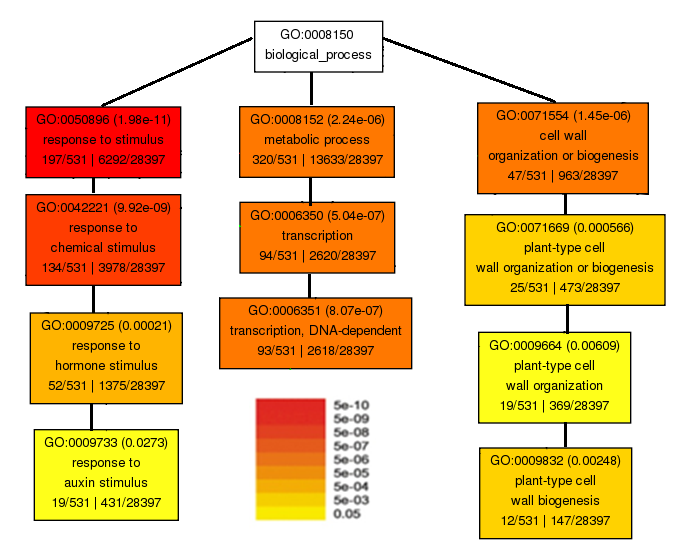


**B)**
