# Supplementary material for: Comparative fiber property and transcriptome analyses reveal key genes potentially related to high fiber strength in cotton (Gossypium hirsutum L.) line MD52ne
Source: BMC Plant Biol. 2016 Feb 1;16:36. doi: 10.1186/s12870-016-0727-2 (PMC4736178; doi:10.1186/s12870-016-0727-2)

Additional file 9. Differential expressions of *receptor like kinases* (*RLK*s) in developing MD52ne fibers at 20 DPA.  Differential exprssions of RLKs in different classes were generated by MapMan. Red and purple color represent up and down regulated, respectively. The RLKs contain three domains including extracellular domain, transmembrane (TM), and kinase domain in a cytoplasmic side. C-lectin, RLKs with lectin-like motifs; Crinkly4-like, RLKs with crinkly4-like domains; DUF26, domain of unknown function 26; Extensin, RLK with extensin motif; L-lectin, RLKs with lectin-binding domains; LRK 10-like, RLK gene on Lr10 locus; LRR, leucine-rich repeats; LysM, RLKs with lysine motif; PERK-like, proline-rich extensin-like kinase; RKF3-like, receptor-like kinase in flowers 3; S-locus, RLK with S-domain; Thaumatin, RLK-like thaumatin protein; WAK, wall-associated kinase.


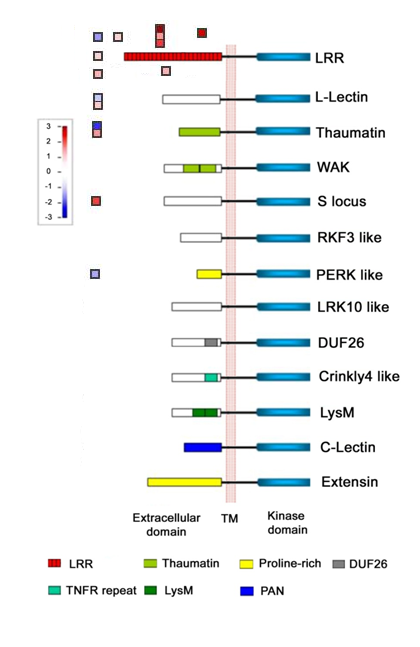

Supplement: Additional file 9: — Differential expressions of receptor like kinases ( RLK s) in developing MD52ne fibers at 20 DPA. Differential expressions of RLKs in different classes were generated by MapMan. Red and purple color represent up and down regulated, respectively. The RLKs contain three domains including extracellular domain, transmembrane (TM), and kinase domain in a cytoplasmic side. C-lectin, RLKs with lectin-like motifs; Crinkly4-like, RLKs with crinkly4-like domains; DUF26, domain of unknown function 26; Extensin, RLK with extensin motif; L-lectin, RLKs with lectin-binding domains; LRK 10-like, RLK gene on Lr10 locus; LRR, leucine-rich repeats; LysM, RLKs with lysine motif; PERK-like, proline-rich extensin-like kinase; RKF3-like, receptor-like kinase in flowers 3; S-locus, RLK with S-domain; Thaumatin, RLK-like thaumatin protein; WAK, wall-associated kinase. This file contains the results of MapMan analysis of different RLKs using differentially expressed genes at 20 DPA. (DOCX 135 kb) [file 12870_2016_727_MOESM9_ESM.docx]
