# Supplementary material for: Comparative fiber property and transcriptome analyses reveal key genes potentially related to high fiber strength in cotton (Gossypium hirsutum L.) line MD52ne
Source: BMC Plant Biol. 2016 Feb 1;16:36. doi: 10.1186/s12870-016-0727-2 (PMC4736178; doi:10.1186/s12870-016-0727-2)

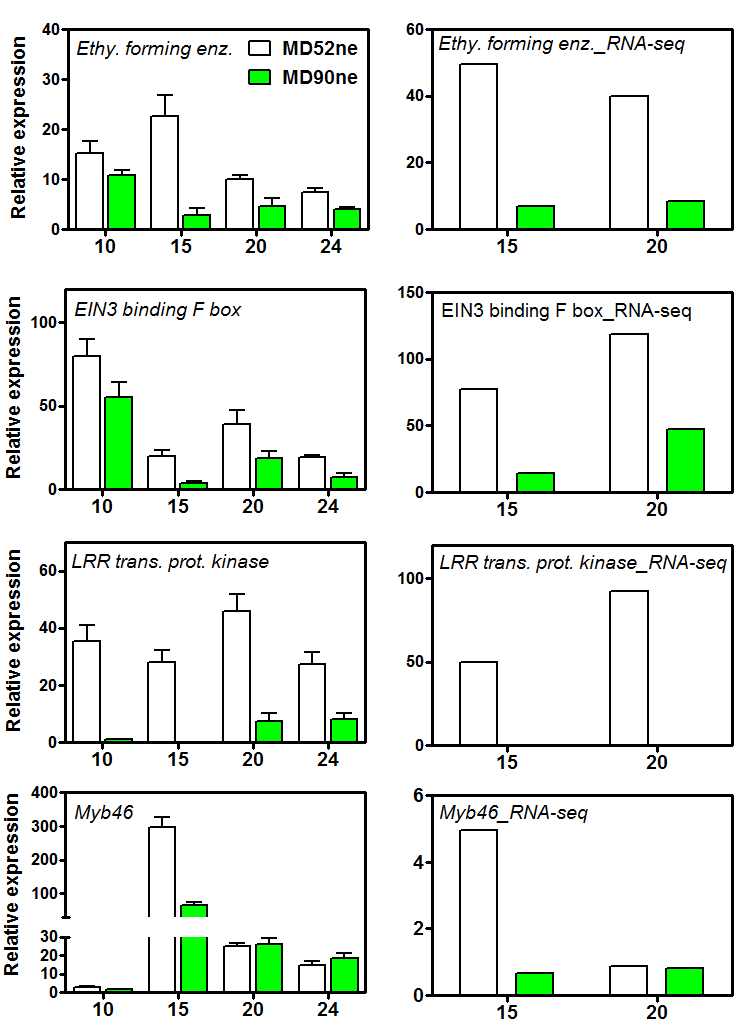
Additional file 10. Quantitative RT-PCR validation of some selected differentially expressed genes from RNA-seq data during fiber development.


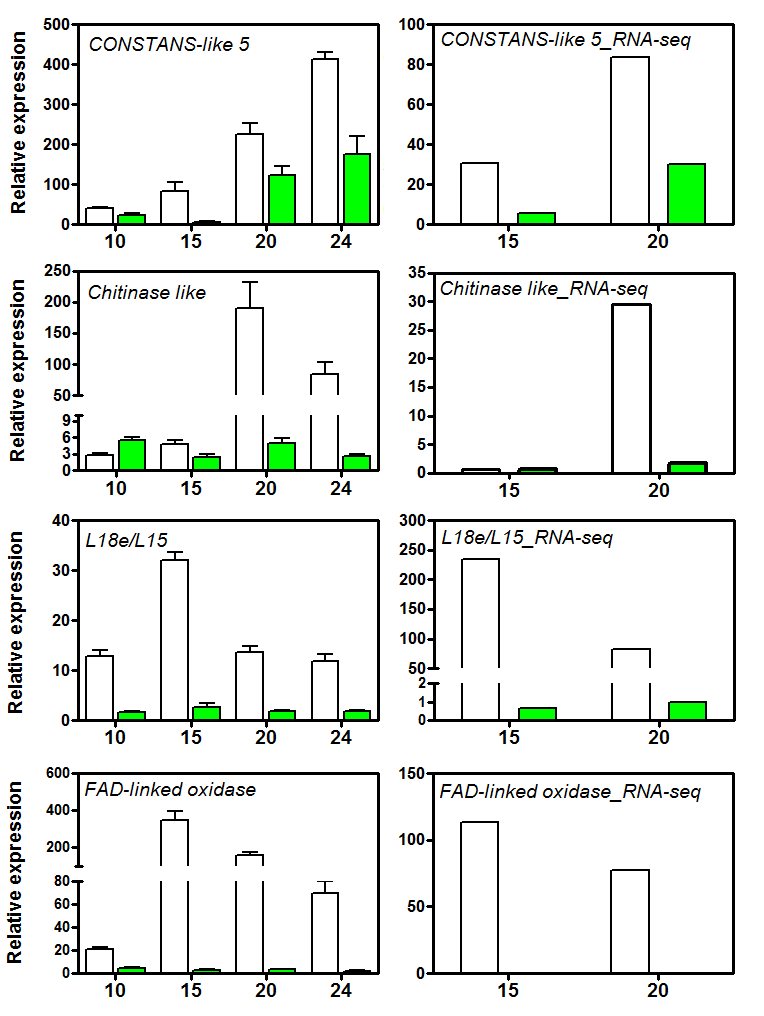


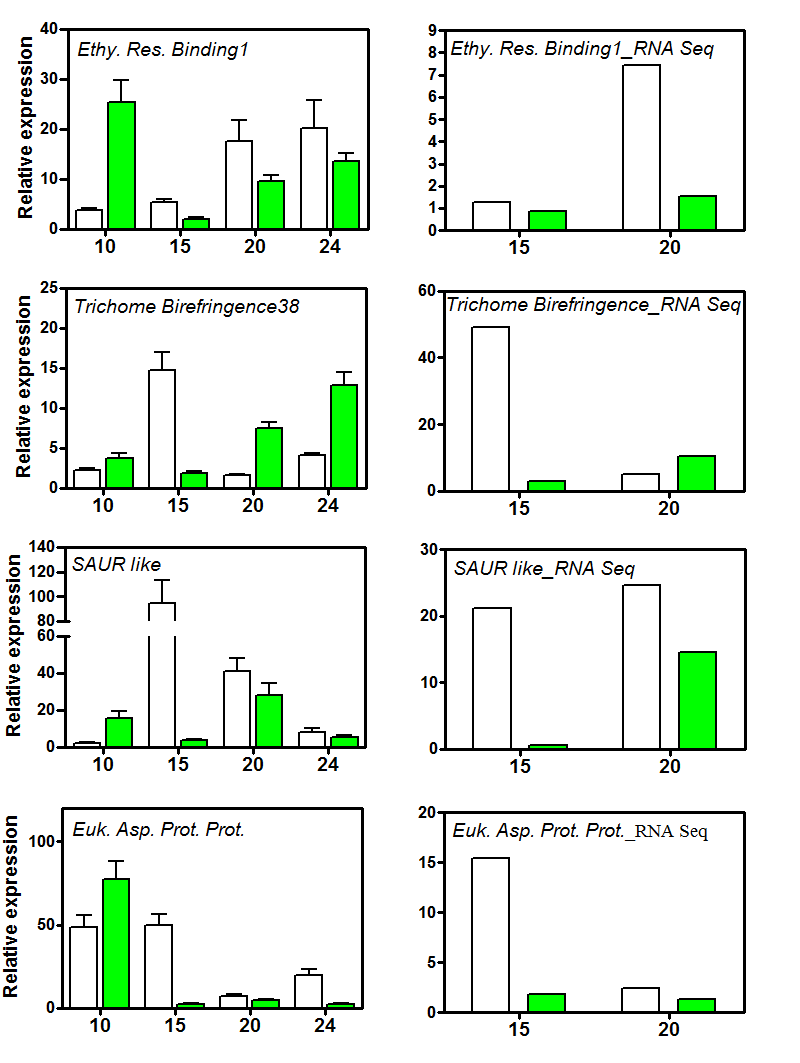

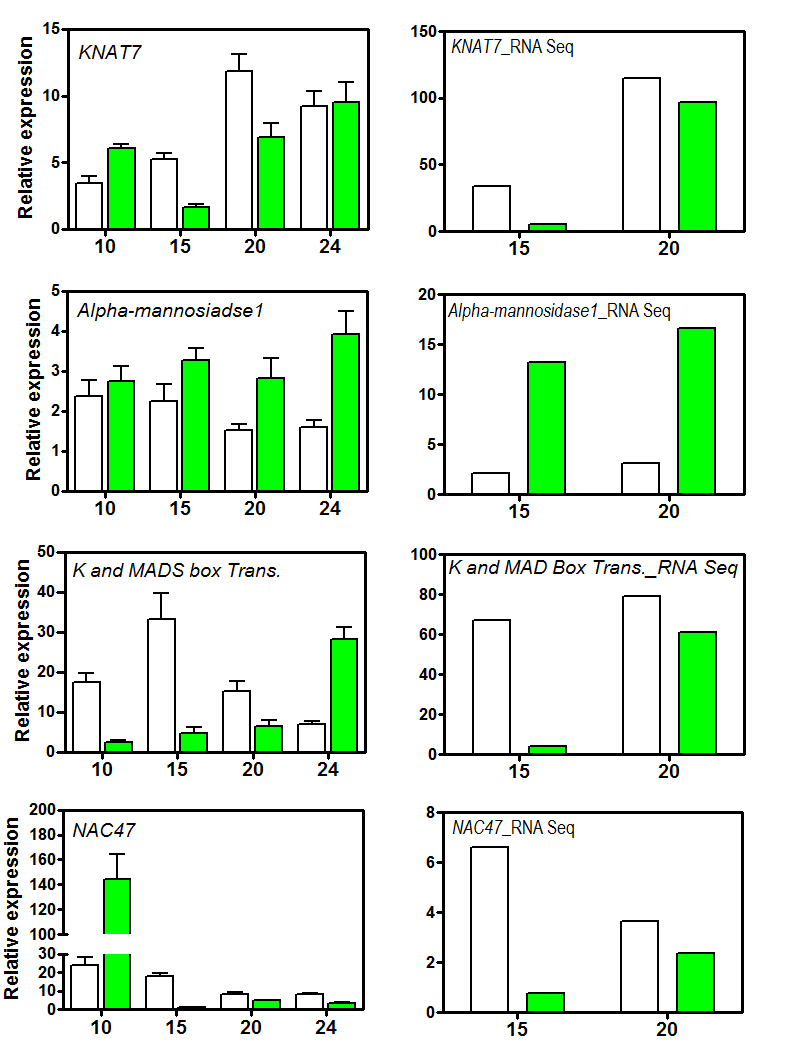


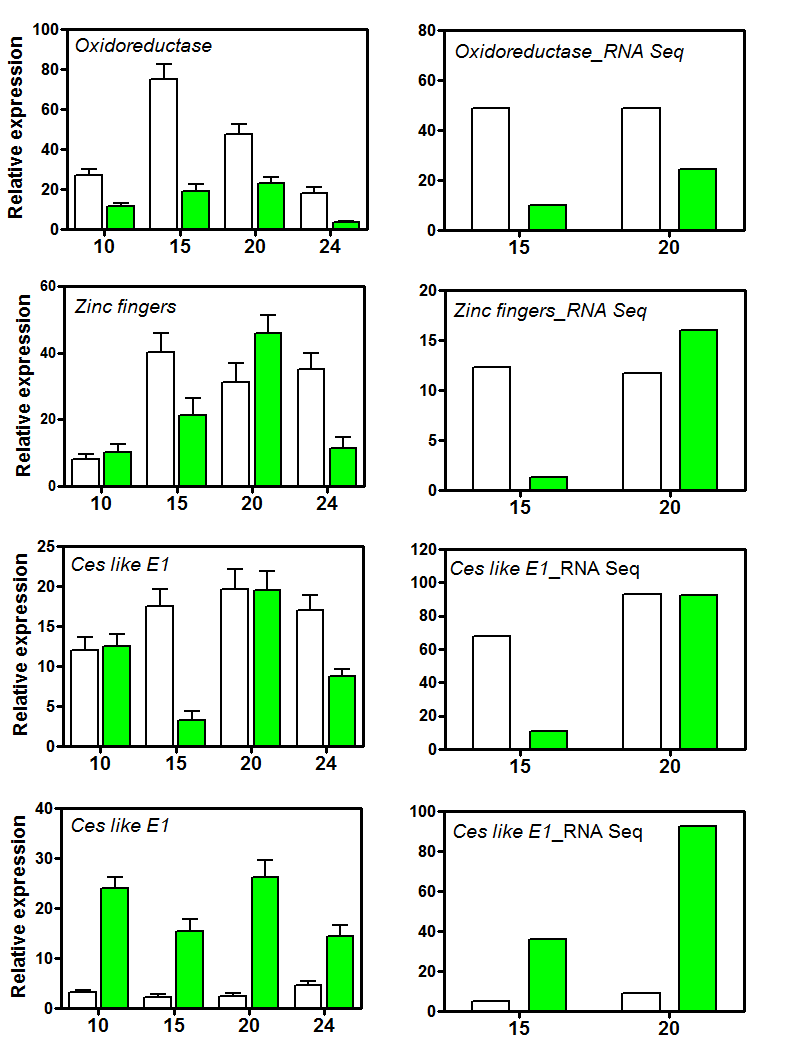


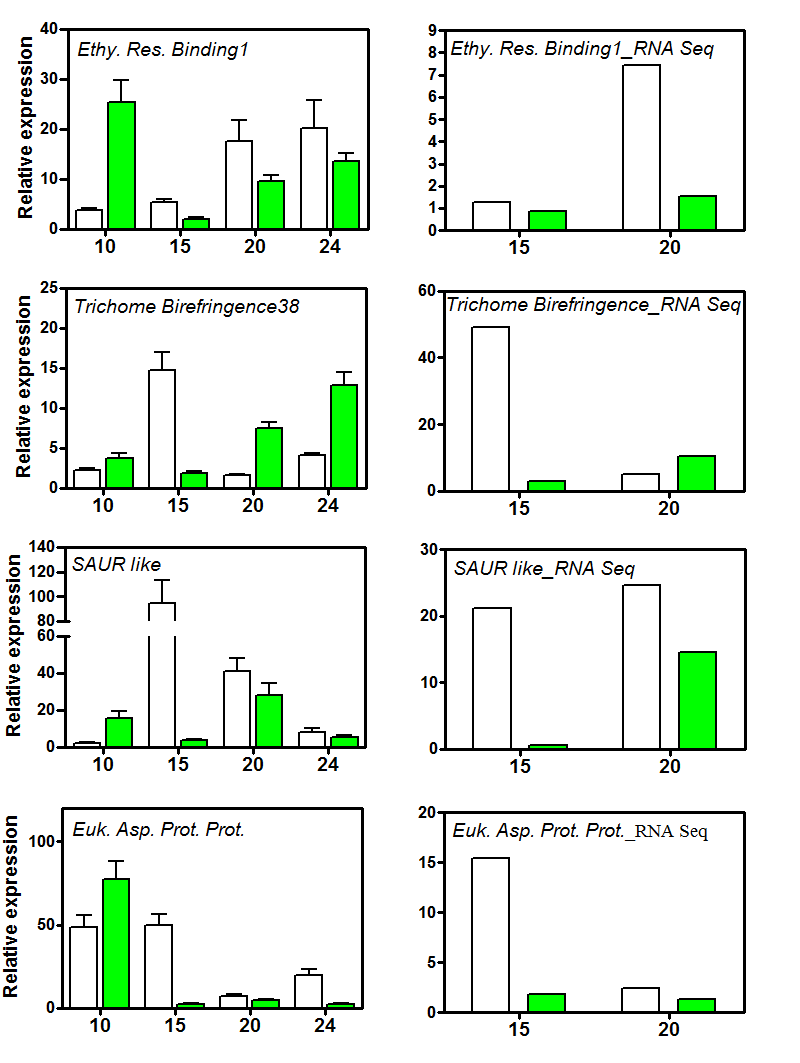


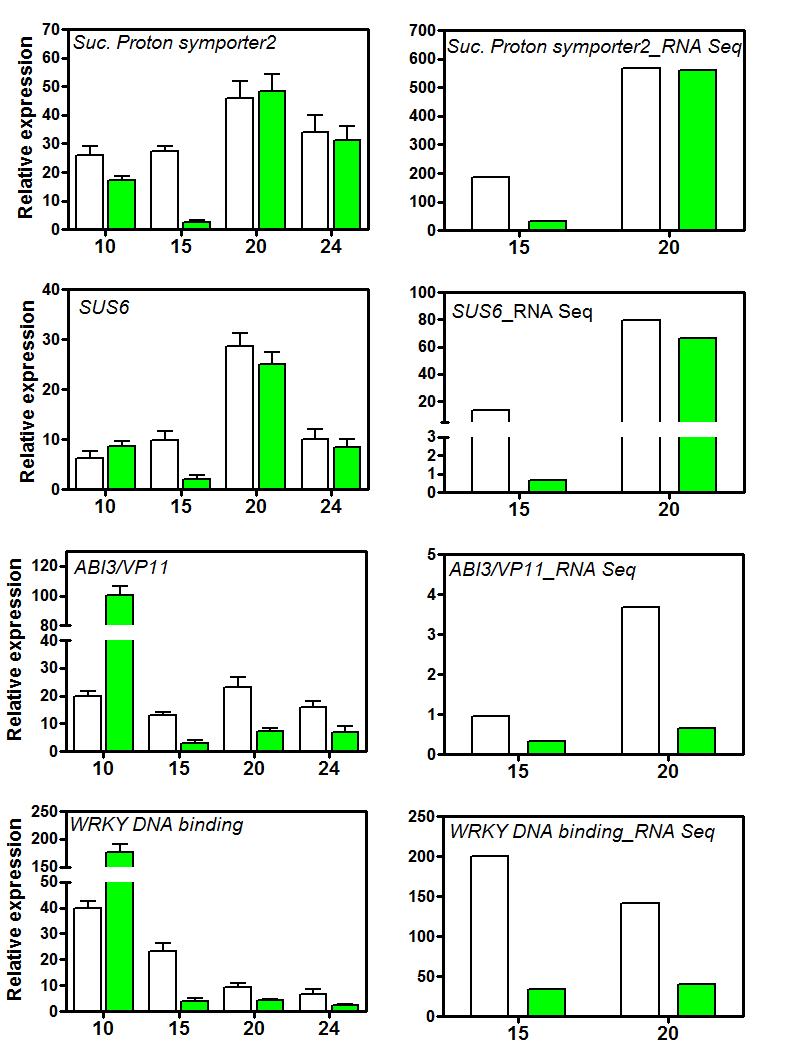


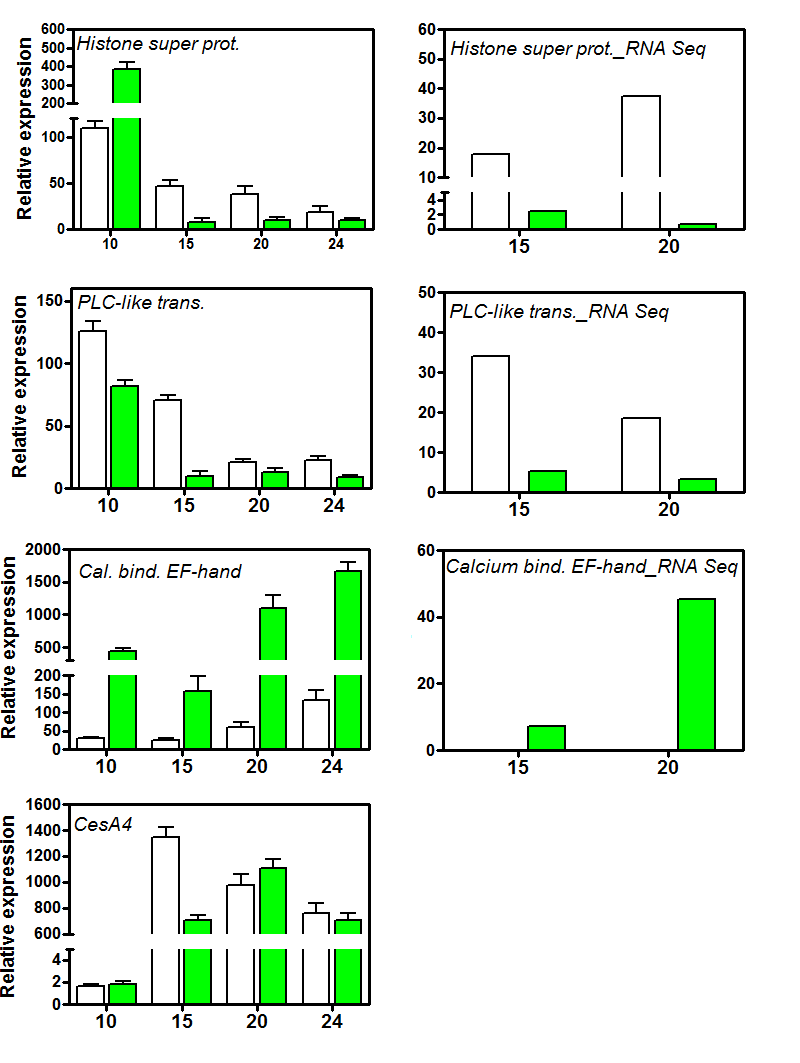

Supplement: Additional file 10: — RT-qPCR validation of some selected differentially expressed genes from RNA-seq data during fiber development. This file contains the results of qPCR analysis and RNA-seq expression data performed on 32 genes that were used to compare fold expression levels between MD52ne and MD90ne. Four (10, 15, 20 and 24 DPA) and two (15 and 20 DPA) developing fiber samples were used for RT-qPCR and RNA-seq, respectively. RT-qPCR values were corrected to Tubilin and 18S gene for each sample. For each treatment group three qPCR measurements were taken for each of three biological replicates and then averaged. (DOCX 644 kb) [file 12870_2016_727_MOESM10_ESM.docx]
