# Supplementary material for: Comparative fiber property and transcriptome analyses reveal key genes potentially related to high fiber strength in cotton (Gossypium hirsutum L.) line MD52ne
Source: BMC Plant Biol. 2016 Feb 1;16:36. doi: 10.1186/s12870-016-0727-2 (PMC4736178; doi:10.1186/s12870-016-0727-2)
Supplement: Additional file 12: — Comparison of deferentially expressed genes between RNA-seq and microarray of Hinchliffe et al. 2010. These figure compare the number of differential expressed genes between RNA-seq (RNA) results of this study and microarray (MA) data reported by Hinchliffe et al. 2010 [17]. RNA-seq and microarray data were generated from 15, 20 and 16, 20 DPA developing fiber samples. A) Total DE genes at both time point; B) DE only expressed in 15 DPA for RNA seq and 16 DPA for microarray data; C) DE only expressed in 20 DPA for both RNA seq and microarray data. (DOCX 320 kb) [file 12870_2016_727_MOESM12_ESM.docx]

Additional file 12. Comparison of deferentially expressed (DE) genes between RNA seq (RNA) data of this study and microarray (MA) data of Hinchliffe et al. 2010. A) Total DE genes at both time point; B) DE only expressed in 15 DPA for RNA seq and 16 DPA for microarray data; C) DE only expressed in 20 DPA for both RNA seq and microarray data.


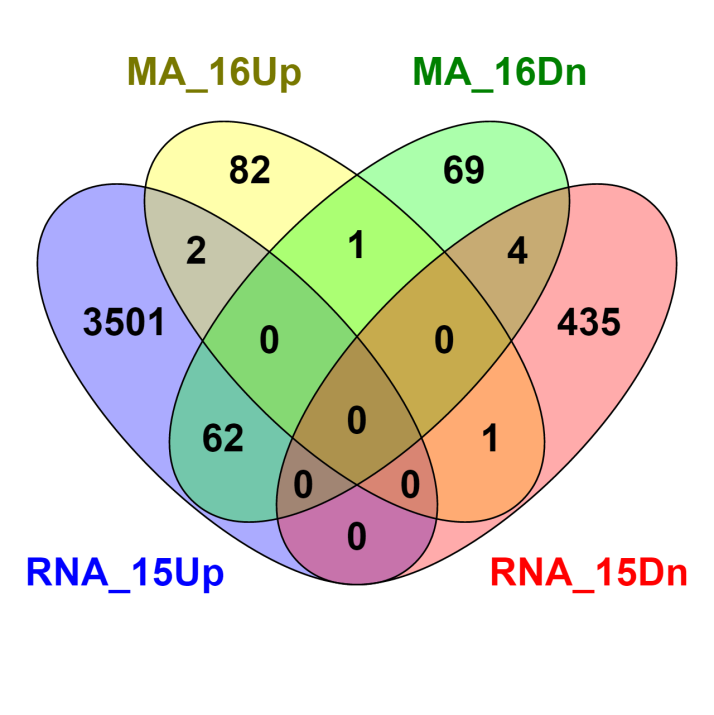

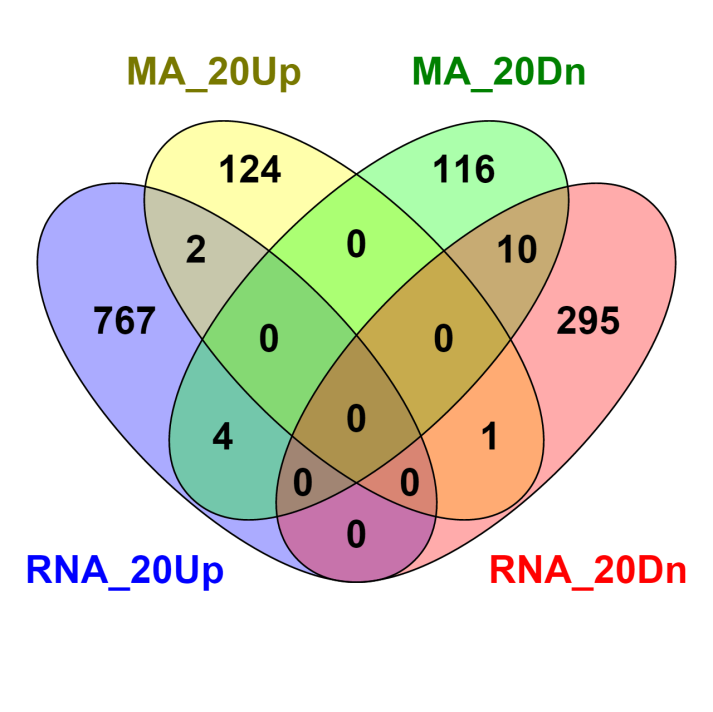

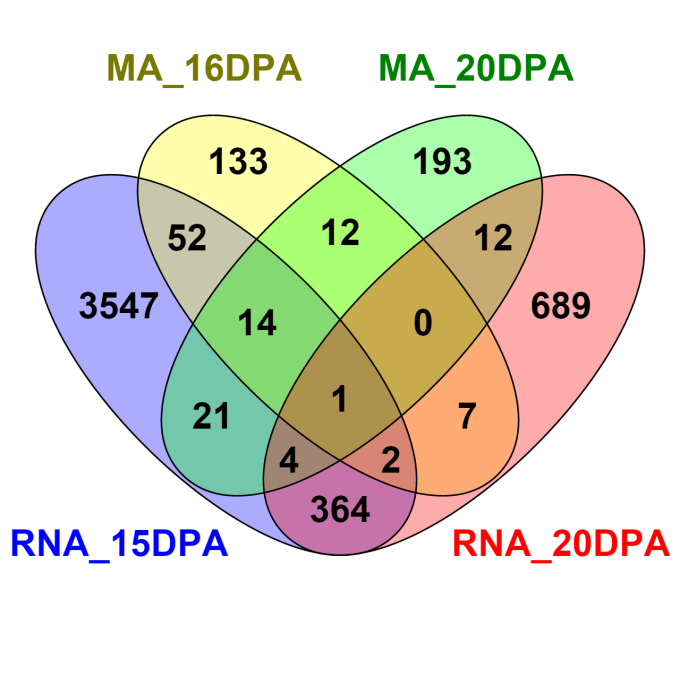


A

B

C
