# Supplementary material for: Comparative fiber property and transcriptome analyses reveal key genes potentially related to high fiber strength in cotton (Gossypium hirsutum L.) line MD52ne
Source: BMC Plant Biol. 2016 Feb 1;16:36. doi: 10.1186/s12870-016-0727-2 (PMC4736178; doi:10.1186/s12870-016-0727-2)
Supplement: Additional file 13: — Cotton MD52ne transcript levels compared with Arabidopsis genes that are co-expressed at secondary wall biogenesis. This table contains 64 secondary cell wall (SCW) biosynthesis related genes retrieved from Arabidopsis genes from PlaNet [67] that are temporally and spatially co-expressed during SCW biosynthesis in Arabidopsis. Those 64 genes were up-regulated in MD52ne. (DOCX 20 kb) [file 12870_2016_727_MOESM13_ESM.docx]

**Additional file 13. Cotton MD52ne transcript levels compared with Arabidospsis genes that are co-expressed at secondary wall biogenesis**

| **No** | **Gene_ID** | **Tair 10 ID** | **Description** | **15 DPA** | **20 DPA** |
| --- | --- | --- | --- | --- | --- |
| 1 | Gh_D07G0819 | AT1G05310 | Pectin lyase-like superfamily protein | 33.87 | 0.75 |
| 2 | Gh_D04G1484 | AT4G35350 | Xylem cysteine peptidase 1 | 15.90 | 1.15 |
| 3 | Gh_D13G1495 | AT5G54690 | Galacturonosyltransferase 12 | 14.72 | 1.29 |
| 4 | Gh_A01G0568 | AT5G54690 | Galacturonosyltransferase 12 | 14.70 | 1.04 |
| 5 | Gh_A10G0858 | AT5G03260 | Laccase 11 | 13.44 | 0.79 |
| 6 | Gh_A06G0019 | AT5G42180 | Peroxidase superfamily protein | 12.66 | 1.09 |
| 7 | Gh_A05G3144 | AT5G14940 | Major facilitator superfamily protein | 11.68 | 1.17 |
| 8 | Gh_D12G2587 | AT2G01940 | C2H2-like zinc finger protein | 11.50 | 1.25 |
| 9 | Gh_A01G1948 | AT2G38080 | Laccase/Diphenol oxidase family protein | 9.89 | 2.22 |
| 10 | Gh_D05G0972 | AT5G60660 | Plasma membrane intrinsic protein 2 | 9.33 | 1.17 |
| 11 | Gh_D03G1128 | AT2G38080 | Laccase/Diphenol oxidase family protein | 9.13 | 1.13 |
| 12 | Gh_A06G1377 | AT4G17220 | Microtubule-associated proteins 70-5 | 9.12 | 1.05 |
| 13 | Gh_A10G0333 | AT1G29200 | O-fucosyltransferase family protein | 8.47 | 0.72 |
| 14 | Gh_A09G1074 | AT5G12870 | MYB domain protein 46 | 7.81 | 1.03 |
| 15 | Gh_D04G1150 | AT2G37460 | Nodulin MtN21 /EamA-like transporter | 7.68 | 0.97 |
| 16 | Gh_D02G1382 | AT2G01940 | C2H2-like zinc finger protein | 7.58 | 1.56 |
| 17 | Gh_A07G1126 | AT5G12870 | MYB domain protein 46 | 6.89 | 3.09 |
| 18 | Gh_D09G0234 | AT5G14940 | Major facilitator superfamily protein | 6.79 | 1.04 |
| 19 | Gh_A08G1366 | AT1G32100 | Pinoresinol reductase 1 | 6.30 | 1.01 |
| 20 | Gh_A13G1199 | AT5G54690 | Galacturonosyltransferase 12 | 6.14 | 1.08 |
| 21 | Gh_D10G0895 | AT5G03260 | Laccase 11 | 5.93 | 0.96 |
| 22 | Gh_A09G0239 | AT5G14940 | Major facilitator superfamily protein | 5.92 | 0.96 |
| 23 | Gh_D12G0779 | AT1G72220 | RING/U-box superfamily protein | 5.86 | 1.48 |
| 24 | Gh_D01G0577 | AT5G54690 | Galacturonosyltransferase 12 | 5.67 | 1.09 |
| 25 | Gh_A10G0599 | AT2G03200 | Eukaryotic aspartyl protease | 5.58 | 1.13 |
| 26 | Gh_A04G0684 | AT2G37460 | Nodulin MtN21 /EamA-like transporter | 5.54 | 0.99 |
| 27 | Gh_A06G1526 | AT5G04200 | Metacaspase 9 | 5.52 | 1.46 |
| 28 | Gh_A11G1031 | AT4G02320 | Invertase/pectin methylesterase inhibitor | 5.27 | 1.03 |
| 29 | Gh_D10G1621 | AT3G21550 | DUF679 domain membrane protein 2 | 5.08 | 0.78 |
| 30 | Gh_D10G0849 | AT5G40020 | Pathogenesis-related thaumatin | 4.92 | 0.69 |
| 31 | Gh_A03G0417 | AT2G38080 | Laccase/Diphenol oxidase family protein | 4.91 | 0.84 |
| 32 | Gh_D11G1187 | AT4G02320 | Invertase/pectin methylesterase inhibitor | 4.76 | 1.52 |
| 33 | Gh_D03G1492 | AT1G62990 | KNOTTED-like homeobox | 4.68 | 1.07 |
| 34 | Gh_D08G1361 | AT3G62020 | Germin-like protein 10 | 4.46 | 1.64 |
| 35 | Gh_D05G2094 | AT1G09890 | Rhamnogalacturonate lyase family protein | 4.45 | 1.16 |
| 36 | Gh_D10G1036 | AT3G18660 | Glycogenin-like starch initiation protein 1 | 4.24 | 0.93 |
| 37 | Gh_A05G1577 | AT5G42180 | Peroxidase superfamily protein | 4.23 | 0.80 |
| **No** | **Gene_ID** | **Tair 10 ID** | **Description** | **15 DPA** | **20 DPA** |
| 38 | Gh_D13G2415 | AT5G60720 | Protein of unknown function, DUF547 | 3.82 | 1.00 |
| 39 | Gh_A07G0672 | AT1G66810 | Zinc finger C-x8-C-x5-C-x3-H type | 3.80 | 2.59 |
| 40 | Gh_A12G2459 | AT2G01940 | C2H2-like zinc finger protein | 3.79 | 0.89 |
| 41 | Gh_A05G3488 | AT3G56230 | BTB/POZ domain-containing protein | 3.66 | 1.06 |
| 42 | Gh_D05G0998 | AT5G60720 | Protein of unknown function, DUF547 | 3.51 | 1.82 |
| 43 | Gh_D08G0310 | AT2G44300 | Lipid-transfer protein | 3.38 | 1.07 |
| 44 | Gh_A04G0239 | AT4G27435 | Protein of unknown function (DUF1218) | 2.98 | 0.95 |
| 45 | Gh_D09G1518 | AT5G02010 | RHO guanyl-nucleotide exchange factor 7 | 2.95 | 0.83 |
| 46 | Gh_A13G2017 | AT5G60720 | Protein of unknown function, DUF547 | 2.85 | 1.03 |
| 47 | Gh_A08G1783 | AT3G45010 | Serine carboxypeptidase-like 48 | 2.81 | 1.24 |
| 48 | Gh_A10G1360 | AT5G01930 | Glycosyl hydrolase superfamily protein | 2.77 | 0.81 |
| 49 | Gh_A13G1084 | AT5G23810 | Amino acid permease 7 | 2.77 | 0.80 |
| 50 | Gh_D11G1449 | AT3G07340 | bHLH DNA-binding protein | 2.70 | 0.93 |
| 51 | Gh_A03G0246 | AT3G45010 | Serine carboxypeptidase-like 48 | 2.67 | 0.89 |
| 52 | Gh_D02G2259 | AT2G16990 | Major facilitator superfamily protein | 2.63 | 0.98 |
| 53 | Gh_A09G1483 | AT2G37460 | Nodulin MtN21 /EamA-like transporter | 2.63 | 1.12 |
| 54 | Gh_D09G1493 | AT2G37460 | Nodulin MtN21 /EamA-like transporter | 2.52 | 1.08 |
| 55 | Gh_D12G1761 | AT5G61430 | NAC domain containing protein 100 | 2.33 | 1.42 |
| 56 | Gh_D09G1082 | AT5G12870 | MYB domain protein 46 | 2.27 | 0.96 |
| 57 | Gh_D05G1462 | AT1G29200 | O-fucosyltransferase family protein | 2.26 | 1.07 |
| 58 | Gh_A12G1620 | AT5G61430 | NAC domain containing protein 100 | 2.24 | 1.20 |
| 59 | Gh_A04G0219 | AT5G54280 | Myosin 2 | 2.22 | 1.83 |
| 60 | Gh_A03G0124 | AT1G63300 | Myosin heavy chain-related protein | 2.20 | 1.00 |
| 61 | Gh_D12G0085 | AT1G03010 | Phototropic-responsive NPH3 family protein | 2.11 | 0.93 |
| 62 | Gh_A12G2076 | AT1G63300 | Myosin heavy chain-related protein | 2.09 | 1.06 |
| 63 | Gh_D10G0339 | AT1G29200 | O-fucosyltransferase family protein | 2.06 | 0.37 |
| 64 | Gh_D01G1332 | AT2G01940 | C2H2 like zinc finger protein | 2.05 | 1.33 |
