# Supplementary material for: Comparative fiber property and transcriptome analyses reveal key genes potentially related to high fiber strength in cotton (Gossypium hirsutum L.) line MD52ne
Source: BMC Plant Biol. 2016 Feb 1;16:36. doi: 10.1186/s12870-016-0727-2 (PMC4736178; doi:10.1186/s12870-016-0727-2)
Supplement: Additional file 14: — Crossing scheme for developing upland cotton near isgenic lines MD52ne and MD90ne. This figure contains crossing scheme for developing upland cotton near isogenic lines MD52ne and MD90ne has taken from Islam et al. 2014 [50]. In parentheses R is for recurrent and D is for donor parent for the respective cross. JCPC, DP, MD and FTA are the germplasm name and stand for John Cotton Poly Cross, Deltapine, Mississippi Delta and ARS strain from Pee Dee experiment station, Florescence, SC. (DOCX 131 kb) [file 12870_2016_727_MOESM14_ESM.docx]

Additional file 14. Crossing scheme for developing upland cotton near isgenic lines MD52ne and MD90ne.
